# RNA-mediated demixing transition of low-density condensates

**DOI:** 10.1038/s41467-023-38118-z

**Published:** 2023-04-27

**Authors:** Taehyun Kim, Jaeyoon Yoo, Sungho Do, Dong Soo Hwang, YongKeun Park, Yongdae Shin

**Affiliations:** 1 Department of Mechanical Engineering, Seoul National University, Seoul, 08826 Republic of Korea; 2 Division of Environmental Science and Engineering, Pohang University of Science and Technology (POSTECH), Pohang, 37673 Republic of Korea; 3 Department of Physics, Korea Advanced Institute of Science and Technology (KAIST), Daejeon, 34141 Republic of Korea; 4 KAIST Institute for Health Science and Technology, Daejeon, 34141 Republic of Korea; 5 Tomocube Inc, Daejeon, 34109 South Korea; 6 Interdisciplinary Program in Bioengineering, Seoul National University, Seoul, 08826 Korea

**Keywords:** Intrinsically disordered proteins, Nuclear speckles, Stress granules, Biopolymers in vivo

## Abstract

Biomolecular condensates play a key role in organizing cellular reactions by concentrating a specific set of biomolecules. However, whether condensate formation is accompanied by an increase in the total mass concentration within condensates or by the demixing of already highly crowded intracellular components remains elusive. Here, using refractive index imaging, we quantify the mass density of several condensates, including nucleoli, heterochromatin, nuclear speckles, and stress granules. Surprisingly, the latter two condensates exhibit low densities with a total mass concentration similar to the surrounding cyto- or nucleoplasm. Low-density condensates display higher permeability to cellular protein probes. We find that RNA tunes the biomolecular density of condensates. Moreover, intracellular structures such as mitochondria heavily influence the way phase separation proceeds, impacting the localization, morphology, and growth of condensates. These findings favor a model where segregative phase separation driven by non-associative or repulsive molecular interactions together with RNA-mediated selective association of specific components can give rise to low-density condensates in the crowded cellular environment.

## Abstract (web summary)

The cell interior is organized by diverse membrane-less condensates. Here, the authors reveal that the densities of certain condensates are surprisingly low, similar to the surrounding protoplasm and driven by cellular RNA as well as the crowded milieu.

## Introduction

The cell uses compartmentalization to organize a suite of complex biological processes. A specific set of biomolecules is enriched inside subcellular compartments or organelles performing diverse cellular functions for proper cell physiology. Some of these intracellular assemblies are membrane-less and are open to the surrounding cyto- or nucleoplasm^1,2^. A liquid-like nature of the membraneless assemblies, also called condensates, led to a hypothesis that liquid-liquid phase separation drives their biogenesis^3^. Over the past several years, numerous works have demonstrated that indeed phase separation plays a key role in the formation of several intracellular condensates^4–9^. Condensates display several biophysical characteristics consistent with the liquid-like material state, such as round morphology, coalescence-mediated growth and dynamic exchange of internal components with the surrounding solution^10,11^. In addition, their assembly is often sensitive to particular stimuli or stresses. This property helps condensates function in diverse cellular settings such as transcription, protein quality control, cell signaling and synapse formation^12–17^. In the perspective of phase separation, the cell interior can be viewed as a multiphase system where distinct condensates with varying compositions coexist in the confined volume.

Molecular driving forces for sorting biomolecules during intracellular phase separation have been extensively studied^18–20^. Weak multivalent molecular interactions mediated by either tandem repeats of interacting domains or intrinsically disordered regions turn out to be important for condensate assembly^13,21,22^. These protein motifs appear to form a network of attractive interactions providing transient intermolecular connectivity within the condensed liquids^23,24^. In purified protein systems, this molecular connectivity manifests as a high concentration of self-associating proteins in the condensed droplets. The dilute phase outside of protein droplets has a much lower protein concentration compared to inside droplets^25,26^, which can cause gravitational sedimentation of protein droplets to the bottom of test tubes. The strength of attractive intermolecular interactions can be tuned by changing conditions such as salt concentrations, pH and crowding reagents, and indeed these parameters have been shown to modulate the phase diagram of purified proteins^21,27^. However, whether attractive molecular interactions alone are enough to give rise to multiphase intracellular condensates is elusive.

The cell interior is a highly crowded environment with an estimated cytosolic protein concentration of 3 mM^28^. Thus, intracellular phase separation is likely affected by the presence of diverse molecular species mediating a broad range of intermolecular interactions including repulsive ones. Notably, a theoretical study of multicomponent phase separation suggests that demixing of a few components from others are favored over condensation as the range of available intermolecular interactions becomes broader^29^. In this classification, condensation involves phases with similar compositions but differing in total concentrations while demixing transition is characterized by a change in composition rather than in total concentration across different phases. Typically, in-vitro phase separation of purified proteins and RNAs, involving dense and dilute phases, is a condensation process in this regard. However, in living cells, the situation can be significantly different. The total protein concentration in the cytoplasm is already close to that in typical in-vitro protein droplets^25,26^. It is thus possible that demixing transition can still provide enough intermolecular connectivity required for condensate formation^23^. This mechanism would then give rise to low-density condensates that have a total mass concentration similar to the surrounding cellular space. Whether this type of phase separation indeed plays a role in the cell remains to be determined.

Here, we examine total mass concentrations of several intracellular condensates, including nucleoli, heterochromatin, nuclear speckles and stress granules. We find that intracellular condensates exhibit a broad range of biomolecular densities, from those with their total mass concentration higher than the surrounding cyto- or nucleoplasm to the low-density ones with their mass densities similar to the outside of the condensate. The low-density condensates are more permeable to cellular protein probes, indicating that the characteristics of low-density is likely to have functional consequences. We provide experimental evidence that RNA tunes the biomolecular density of intracellular condensates. We also show that intracellular structures heavily influence the way condensates form and distribute within the cell. These findings suggest that the crowded intracellular environment is an important factor shaping the structure and dynamics of biomolecular phase separation in living cells.

## Results

### Nucleoli and heterochromatin are denser than the surrounding nucleoplasm

To quantify a total concentration of biomolecules, total mass concentration, within condensates, we measure the three-dimensional (3D) distribution of refractive index (RI), *n*, in living cells. Over several decades, the RIs of a solution of biomolecules have been shown to linearly proportional to the concentration of biomolecules^30,31^. The increments of RI with respect to concentration (dn/dc) for most biomolecules including proteins and nucleic acids fall within a narrow range^31–34^, enabling the estimation of total mass concentrations, or mass densities, based on RI measurements. Indeed, the RI imaging has been widely used to profile the local dry mass density distribution within the cell^31,35,36^ as well as to characterize the concentration of purified proteins in phase-separated liquid droplets^37^. The RI increment in principle depends on specific sequence compositions^38^, which can give rise to different RI increments for distinct proteins. However, as inherently multi-component systems, intracellular condensates typically harbor hundreds of different components. Using available proteomes^39–41^, we estimate that the RI increments of condensate components are similarly distributed to the entire human proteome (Fig. S1a), indicating that heterogeneity in the protein RI increments will be averaged out during the diffraction-limited RI imaging. To measure the intracellular distribution of RIs, we employ optical diffraction tomography (ODT)^42–44^. In the ODT, the 3D RI tomogram is reconstructed from 2D optical field images acquired from multiple illumination angles (see Methods)^43^. Unlike 2D phase imaging techniques which measure the line integration of RI values over an optical path, the ODT offers a key advantage for probing intracellular condensates in that the separate characterization of condensate morphology is unnecessary (Fig. S1b–e). A similar approach has been taken in several studies to measure the RI of subcellular regions, including nucleoli, mitochondria, nucleoplasm and cytoplasm^45–47^.

**Fig. 1:**
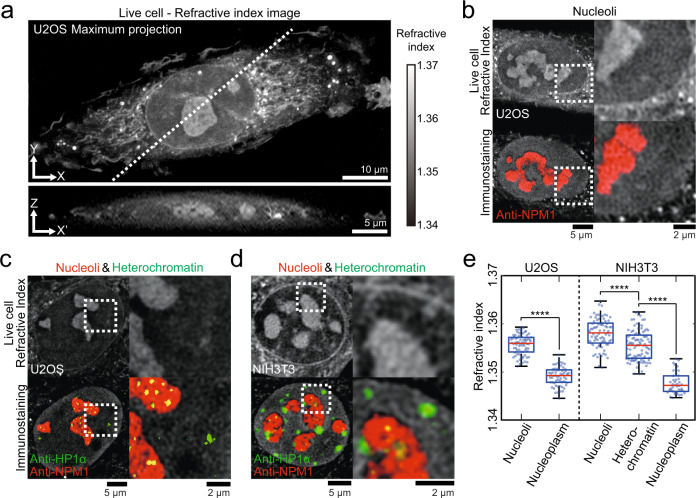
Quantitative refractive index imaging of intracellular condensates. **a** (Top) Maximum intensity projection of the 3D RI tomogram of an intact live U2OS cell. (Bottom) Cross-sectional view of the same cell along a white dashed line. **b**–**d** (Top row) RI images of intact live cells. (Bottom row) Combined RI and immunofluorescence images of the same cell after fixation. The dashed boxes indicate areas for zoomed-in images on the right. **b** RI images of an intact live U2OS cell and the same cell after immunostaining with anti-NPM1. **c** RI images of an intact live U2OS cell and the same cell after double-immunostaining with anti-NPM1 and anti-HP1α. **d** RI images of an intact live NIH3T3 cell and the same cell after double-immunostaining with anti-NPM1 and anti-HP1α. **e** Quantification of refractive indices of intracellular regions in intact live cells. Data: center line = mean; box limits = [Q1, Q3]; whiskers = [Max, Min]; *n* = 73 (U2OS Nucleoli), 62 (U2OS Nucleoplasm), 79 (NIH3T3 Nucleoli), 98 (NIH3T3 Heterochromatin) and 42 (NIH3T3 Nucleoplasm). Distributions were statistically compared using the two-sided unpaired t-test. *p* = 1.83 · 10^−39^; *p* = 9.63 · 10^−8^; *p* = 5.03 · 10^−32^; ****p < 0.0001. Refractive index images are adjusted to the range of refractive index 1.34–1.37 for (**a**) and (**d**) and 1.337–1.37 for (**b**) and (**c**).

Using ODT, we sought to measure the RI of biomolecular condensates in living cells. When we perform RI imaging of live intact U2OS cells, it is immediately clear that in nucleus there are visible structures resembling nucleoli (Fig. 1a). To confirm this, we fix the cell and perform immunostaining using an antibody against NPM1, a major protein component of nucleoli. We then locate the very same cell we imaged in its live state and then perform combined fluorescence and RI imaging (Fig. 1b and Fig. S2a). Comparing these images, we confirm that indeed the majority of visible nuclear structures in the RI images corresponds to nucleoli. Consistent with previous works^46,47^, these data indicate that nucleoli have a higher total mass concentration compared to the surrounding nucleoplasm.

A closer examination of the RI images shows other identifiable structures in the nucleus that are not stained with NPM1 (Fig. 1b and Fig. S2a). The nuclear periphery also exhibits higher RI values than nucleoplasm (Fig. 1a). Heterochromatin is a set of condensed chromatins mostly comprised of repetitive genes in the silenced state, and thought to assemble via phase separation of DNA and DNA-binding proteins such as HP1^7,48^. Heterochromatin domains appear as small foci distributed around nucleoplasm, but are often found near nucleoli and nuclear laminar. We reason that the dense nuclear structures devoid of NPM1 signals can be heterochromatin domains. To confirm this, we perform live-cell RI imaging followed by immunofluorescence targeting the heterochromatin marker HP1α. Indeed, we find that the nuclear structures of high biomolecular density yet devoid of NPM1 correspond to heterochromatin (Fig. 1c and Fig. S2b). Since heterochromatin domains in U2OS cells tend to be too small for reliable quantification of their RI values, we image NIH3T3 cells that are known to have particularly large heterochromatin called chromocenters (Fig. S2c). In NIH3T3 cells, as expected, large heterochromatin domains with RIs higher than nucleoplasm are clearly visible, distinct from nucleoli^49^ (Fig. 1d and Fig. S2d, e). We repeat these procedures for multiple cells and characterize the RIs of nucleoli, heterochromatin, and nucleoplasm (Fig. 1e): the estimated total mass concentrations, or mass densities, are 87.71 ± 2.07 mg/ml for nucleoli in U2OS, 53.31 ± 1.62 mg/ml for nucleoplasm in U2OS, 100.36 ± 2.99 mg/ml for nucleoli in NIH3T3, 86.84 ± 3.1 mg/ml for heterochromatin in NIH3T3, and 45.84 ± 2.2 mg/ml for nucleoplasm in NIH3T3 cells. We observe that nucleoli tend to have higher RIs than heterochromatin. Taken together, these data show that nucleoli and heterochromatin are dense structures with total mass concentrations higher than the surrounding nucleoplasm.

### Nuclear speckles and stress granules are low-density condensates

**Fig. 2:**
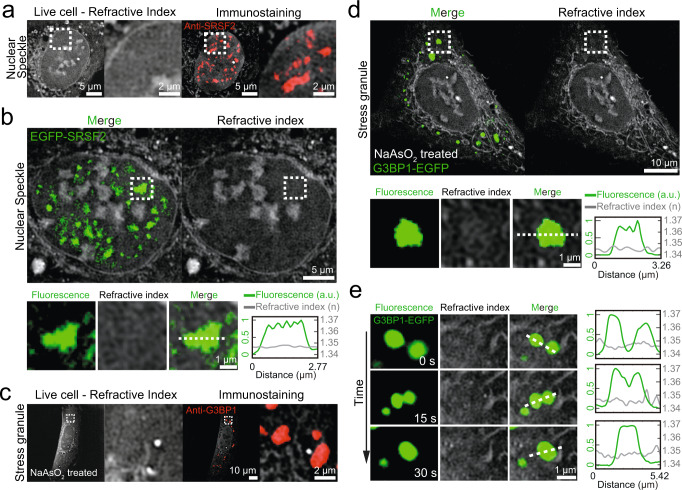
Nuclear speckles and stress granules are low-density condensates. **a** (Left) RI images of an intact live U2OS cell. (Right) Combined RI and immunofluorescence images of the same cell after fixation. Anti-SRSF2 is used to target nuclear speckles. **b** (Top) Combined RI and fluorescence images of a live U2OS cell expressing EGFP-SRSF2. (Bottom left) Zoomed-in images of a single nuclear speckle. (Bottom right) Intensity profile plot for normalized fluorescence intensities of EGFP-SRSF2 (green line) and RIs (gray line) along the white dashed line. **c** (Left) RI images of a live U2OS cell treated with 500 μM sodium arsenite. (Right) Combined RI and immunofluorescence images of the same cell after fixation. Anti-G3BP1 is used to target stress granules. **d** (Top) Combined RI and fluorescence images of a live U2OS cell expressing G3BP1-EGFP after the treatment of 500 μM sodium arsenite. (Bottom left) Zoomed-in images of a single stress granule. (Bottom right) Intensity profile plot for normalized fluorescence intensities of G3BP1-EGFP (green line) and RIs (gray line) along the white dashed line. **e** (Left) Time-lapse images of a fusion event between two stress granules. (Right) Intensity profile plots for normalized fluorescence intensities of G3BP1-EGFP (green line) and RIs (gray line) along white dashed lines. All refractive index images are adjusted to 1.337–1.37.

Nuclear speckles are large condensates found in the nucleus and thought to be involved in transcription and RNA processing^50^. Given their large size reaching up to a few micrometers, it should be straightforward to find them in images if they are present. Interestingly, however, we cannot identify any signature of nuclear speckles in RI images we examined (Fig. 1b–d). This suggests that nuclear speckles may have RIs similar to the surrounding nucleoplasm, and thus not identifiable. To test this, we perform live-cell RI imaging and immunofluorescence with SRSF2, a marker protein for nuclear speckles. After identifying nuclear speckles in the immunofluorescence images, we examine the corresponding regions in the live-cell RI data to check the presence of any structures. Indeed, no structure is visible in the regions (Fig. 2a and Fig. S3a). A caveat of this experiment is that intracellular structures under scrutiny may move during fixation. To rule out this possibility and validate our observation, we express EGFP-tagged SRSF2 in the U2OS cell. Combined fluorescence and RI imaging of live cells expressing EGFP-SRSF2 shows that nuclear speckles are indeed invisible in the RI images, indicating that they have a density similar to the surrounding nucleoplasm, but much lower than dense condensates such as nucleoli and heterochromatin (Fig. 2b and Fig. S3b; Supplementary Movie 1).

We wonder whether the presence of the low-density condensate is specific to the nucleoplasm. Stress granules are large cytoplasmic condensates that dynamically assemble under stressful conditions^51^. We sought to probe the biomolecular density of stress granules. After inducing stress granule formation by treating cells with 500 μM sodium arsenite, we perform live-cell RI imaging followed by immunofluorescence with G3BP1, a marker for stress granule (Fig. 2c and Fig. S4a). Surprisingly, no visible structure is found in the RI images in the cytoplasmic regions corresponding to G3BP1-positive stress granules (Fig. 2c). We confirm our observation using fluorescent protein tagged G3BP1 (Fig. 2d and Fig. S4b; Supplementary Movie 2). When examined for two different cell types, NIH3T3 and U2OS cells, stress granules are indistinguishable from the surrounding cytoplasm in the RI images except for a small population (< 18.5%) in U2OS cells (Fig. S5a, b). Stress granule formation is a highly dynamic process involving a series of fusion events between condensates. While individual fusions are clearly visible in the fluorescence channel, they are completely absent in the corresponding RI images (Fig. 2e), further confirming the low-density nature of stress granules. Together, our data provide direct evidence showing that there exist low-density intracellular condensates of which density are not so much different from the surrounding cyto- and nucleoplasm.

### Low-density condensates have highly porous and permeable internal organization

**Fig. 3:**
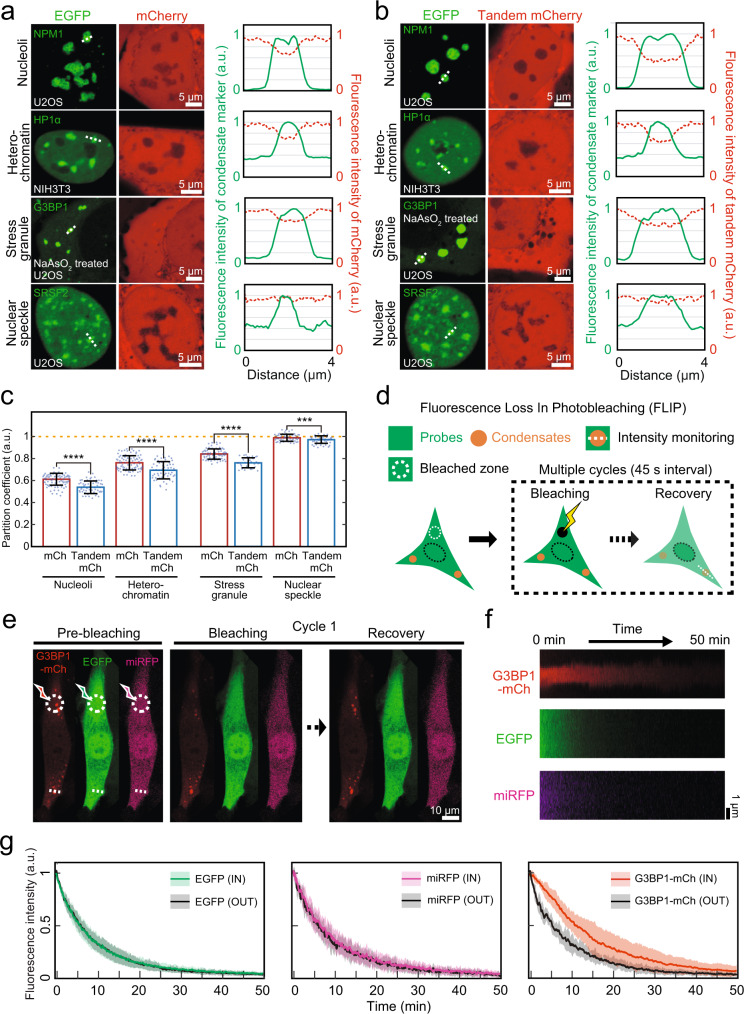
Low-density condensates have highly porous and permeable internal organization. **a**, **b** Representative images of U2OS cells stably expressing EGFP tagged NPM1, G3BP1, and SRSF2 and NIH3T3 cell stably expressing EGFP-HP1α. All cells are co-transduced with mCherry in (**a**) or tandem mCherry (mCh-mCh) in (**b**). G3BP1-EGFP expressing cells are treated with 500 μM sodium arsenite. For each condensate indicated with white dashed lines, intensity profile plots for normalized fluorescence of EGFP-tagged proteins and mCherry probes were shown in solid and dashed lines, respectively. **c** Partitioning coefficients of mCh and tandem mCh probes for each condensate type. Data: center line = mean; whiskers = [mean + std, mean - std]; *n* = 79, 86, 70, and 52 (mCh, respectively) 57, 57, 34, and 39 (tandem mCherry, respectively). Distributions were statistically compared using the two-sided unpaired t-test. *p* = 1.19 · 10^−8^; *p* = 1.67 · 10^−8^; *p* = 2.36 · 10^−13^; *p* = 1.56 · 10^−4^; ****p < 0.0001 and ***p < 0.001. **d** Schematic for the FLIP experiment. After acquiring 5 images for normalization, 100 cycles, each comprised of one bleaching event and 15 image acquisition, are followed. White dashed lines indicate the region where intensity profiles are measured for each image. **e** Time-lapse images of an NIH3T3 cell treated with 250 μM sodium arsenite during the FLIP experiment. The cell is expressing G3BP1-mCh, EGFP and miRFP. For every cycle, a bleaching event occurs at the white dashed circle for all three fluorescence channels. The white dashed line indicates the stress granule across which fluorescence changes are monitored over time. **f** Kymographs showing fluorescence changes in each channel, generated along the white dashed line in (**e**). **g** Temporal changes of fluorescence intensity of protein probes (left and middle) or G3BP1-mch (right) in and outside of the stress granule in (**f**). *n* = 3. The average lines were computed with a sliding window of 5 frames (15 s). Error bands denote standard deviations.

The low-density nature of condensates suggests that they have highly porous internal organization where biomolecules may easily access. To test this idea, we use fluorescent proteins, mCherry and tandem dimer of mCherry (mCh-mCh), as probes, and measure their accessibility to four different types of condensates we examined. We hypothesize that mCherry probes would exhibit size-dependent exclusion from condensates. We co-express mCherry probes and EGFP-tagged condensate markers, and then image cells using laser scanning confocal microscopy (Fig. 3a and Fig. S6a). We observe that denser condensates such as nucleoli and heterochromatin tend to exclude probe molecules, consistent with previous works^52^. However, we find that low-density condensates such as nuclear speckles and stress granules are more accessible to the probes. Linear intensity profiles confirm that fluorescent protein probes are more evenly distributed across the boundary of low-density condensates, in particular, nuclear speckles. Moreover, consistent with the idea of size-dependent exclusion, the larger tandem mCherry probes are excluded more strongly, regardless of condensate type. (Fig. 3b and Fig. S6a) For each type of condensates, we also quantify partition coefficients of the probe into the condensate (Fig. 3c and Fig. S6b), defined as the ratio of the probe concentration in the condensate versus the surrounding protoplasm. To rule out the possibility of underestimating partition coefficients from the small size of condensates, we only measured coefficients for condensates larger than 1 μm. We again find out that low-density condensates have partition coefficients near unity but high-density ones have systematically lower partitioning of the probes.

To further study the molecular dynamics of the probe within condensates, we perform fluorescence loss in photobleaching (FLIP) experiments. In FLIP, a subcellular region of the cell is photobleached while monitoring fluorescence intensity changes in the region of interest away from the bleached zone (Fig. 3d). The assay can be used to characterize the dissociation or diffusive efflux of molecules away from the structures of interest^53^. We prepare U2OS cells expressing G3BP1-mCh, EGFP and miRFP, and treat them with sodium arsenite to induce stress granule assembly. We then locally illuminate bleaching lasers on a small area at the corner of the cell while monitoring fluorescence signals around one of stress granules located near the opposite side of the cell (Fig. 3e). In our illumination conditions, we confirm that fluorescence signals in neighboring cells remain unaffected during the FLIP experiments, indicating that fluorescence decreases in the region of interest are not due to direct bleaching effects, but rather originated from diffusive efflux (Fig. S6c, d). To examine the rate of efflux of fluorescent protein probes as well as G3BP1 from stress granules, we generate kymographs across the stress granule (Fig. 3f). We find that fluorescence levels of the protein probes in the stress granule decrease at a similar rate to those in the surrounding cytoplasm, indicating that they explore the internal space of stress granules with similar mobility as they do in the cytoplasm. (Fig. 3g) In contrast, G3BP1 exhibits slower efflux from stress granules, which is attributable to attractive intermolecular interactions between G3BP1 and other stress granule components^24^. Thus, our data suggest that low-density condensates have highly porous internal architectures where cellular proteins can access.

### RNA tunes the biomolecular density of condensates in cells

We then sought to identify a major determinant for the low-density nature of condensates. Multiple lines of evidence suggest that RNA likely plays an important role in driving the assembly of low-density condensates such as stress granules and nuclear speckles. First, RNAs are highly abundant in these condensates, as examined with poly-dT FISH^54,55^. Second, RNAs are important for the biogenesis of these condensates; tethering RNA can seed nuclear speckle assembly^56^, and stress granules form when the excess of free mRNA becomes available due to ribosome run-off in the stress response^51^ or transfection of mRNAs into cells^57^. Third, the amount of poly(A)-positive RNA in individual nuclear speckles showed a strong correlation with speckle size^58^. Finally, in-vitro studies with purified protein components of low-density condensates demonstrated that droplets comprised of both protein and RNA had much lower total mass density compared to protein-only ones^59^. Thus, diverse RNA species present in nuclear speckles and stress granules may help form the condensates of low-density.

**Fig. 4:**
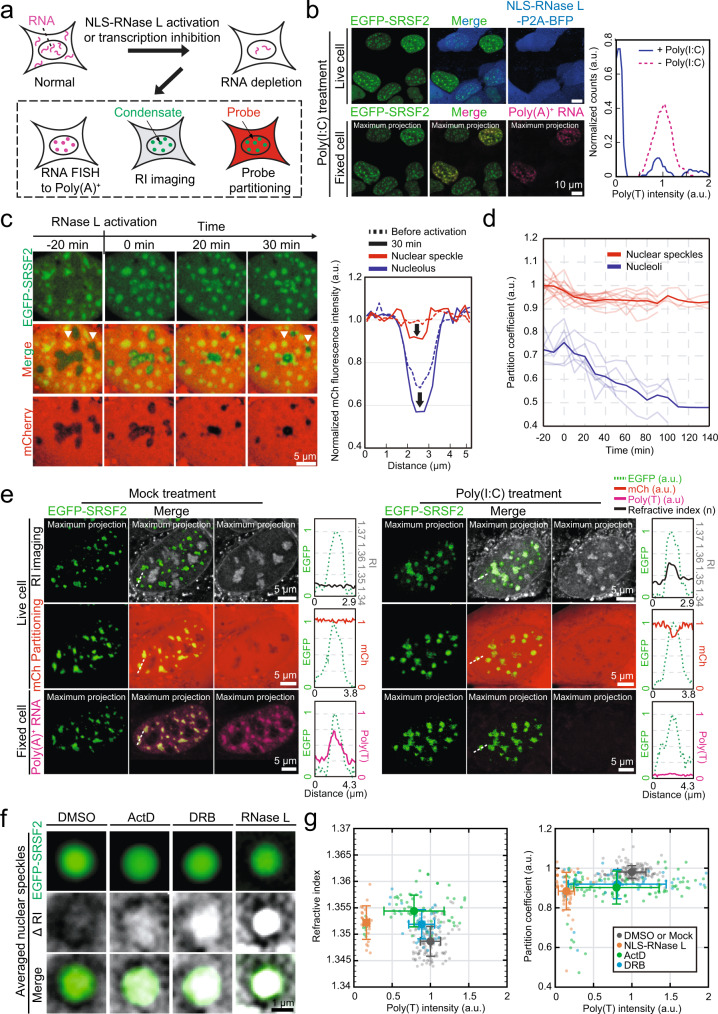
RNA tunes the biomolecular density of intracellular condensates. **a** Schematics of RNA depletion experiments. After activating NLS-RNase L by poly(I:C) treatment or treating cells with either DRB or ActD, a series of quantitative characterizations were conducted. **b** (Left) Representative images of U2OS cells stably expressing EGFP-SRSF2 and NLS-RNase L-P2A-BFP after poly(I:C) treatment. After live-cell images were collected, poly-dT RNA FISH images were acquired for the same cells. (Right) Distribution of poly-dT intensities within individual nuclear speckles. Only cells expressing both constructs were included. The normalized counts were averaged with a sliding window of 0.2 (a.u.). *n* = 146 (mock) and 119 (poly(I:C)) **c** (Left) Time-lapse images of a live U2OS cell stably expressing EGFP-SRSF2, mCherry, and NLS-RNase L after poly(I:C) treatment. Time 0 is defined when the nuclear speckle morphology begins to change. (Right) Normalized intensity profiles for mCherry across either a nuclear speckle or a nucleolus labeled with arrowheads in (**c**; Left). **d** Temporal changes of the partition coefficients of mCherry for nuclear speckles or nucleoli in U2OS cells after NLS-RNase L activation. Time is defined as in (**c**). The bold lines denote average values. *n* = 14 (nuclear speckles) and 6 (nucleoli). **e** Representative images of U2OS cells expressing EGFP-SRSF2, mCherry, and NLS-RNase L-P2A-BFP under mock treatment (Left) or poly(I:C) treatment (Right). After imaging live-cells, poly-dT RNA FISH images were acquired for the same cells. Normalized intensity profiles of EGFP-SRSF2 (green), RI (gray), mCh (red), and poly-dT (pink) along white dashed lines are shown. RI images are adjusted to the range of 1.34–1.37. **f** Averaged RI and fluorescence images of nuclear speckles for each RNA depletion condition. Individual images of nuclear speckles (same datasets as in **g**; Left) were center-aligned using fluorescence signals before averaging. ΔRI images were obtained by subtracting the minimum RI pixel value of each averaged RI image and adjusted to the range of 0–0.0025. **g** For individual nuclear speckles, either refractive indices (Left) or partition coefficients of mCherry (Right) were plotted against poly-dT intensities. Each experimental condition is color-coded: gray (DMSO or Mock), orange (NLS-RNase L), green (ActD), and blue (DRB). Data: center dot = mean; whiskers = [mean-std, mean+std].

To probe the effect of RNA on condensate density, we deplete cellular RNA by treating U2OS cells with 5,6-dichloro-benzimidazole riboside (DRB) or Actinomycin-D (ActD), which inhibits transcription through disruption of CDK9-mediated pause release of RNA Pol II and DNA intercalation, respectively^60,61^ (Fig. 4a). For ActD-treated cells, we find that oxidative stress conditions fail to induce stress granule formation^57^ (Fig. S7a), confirming the critical role of RNAs in stress granule assembly. In contrast, nuclear speckles persist after ActD or DRB treatment, consistent with previous results^50,62^ (Fig. S7b). Interestingly, the speckles adopt more spherical morphologies after chemical treatment (Fig. S7b), implying a change in the internal organization of nuclear speckles upon inhibition of RNA influx. To quantify the degree of RNA depletion, we performed RNA fluorescence in situ hybridization (FISH) targeting poly(A)-positive transcripts for DMSO- and DRB/ActD-treated cells. In the control DMSO-treated sample, we find that nuclear speckles are highly enriched in poly(A) RNAs as expected^54^ (Fig. S7c). Upon DRB or ActD treatment, we observe a strong heterogeneity in the amount of RNA in nuclear speckles, with 23.2% and 26.5% of reduction on average, respectively (Fig. S7d). We then ask whether the decrease in the nuclear RNA level affects either probe accessibility or the biomolecular density of nuclear speckles. Considering the intercellular variation in the level of remained RNA, we collected poly-dT FISH data for cells where probe partitioning or refractive indices were quantified. Strikingly, we find that, in contrast to the untreated and DMSO-treated cases, nuclear speckles with reduced poly(A)-positive transcripts are often identifiable in the RI imaging (Fig. S7e, f), indicating that, upon RNA depletion they become denser than the surrounding nucleoplasm. Consistent with a decrease in internal space available to cellular proteins, we observe that mCherry probes become excluded from nuclear speckles in the DRB/ActD-treated cells (Fig. S7g).

Since RNA depletion through transcription inhibition leads to strong cell-to-cell variability and often incomplete reduction in nuclear poly(A) RNA, we sought to use an alternative approach for rapid removal of cellular RNA. Recently, activating NLS-tagged RNase L with poly(I:C), a viral dsRNA mimic, has been shown to be an effective way of depleting nuclear RNA^63,64^. Upon treating NLS-RNase L expressing cells with poly(I:C), we observed a near complete depletion (4.5% remained) of nuclear poly(A) RNA (Fig. 4b). RNase L activation triggers abrupt changes in nuclear speckle morphologies as well as SRSF2 partitioning (Fig. 4c, Fig S8a, and Fig. S9a, b). In response to poly(I:C) treatment, the shape of nuclear speckles changes from irregular- to spherical ones while frequent fusions between neighboring speckles are observed (Fig. S8a and Fig. S9a, b). Concurrently, a subpopulation of SRSF2 partitions into nucleoli which exhibit a drastic reduction in size (Fig. 4c, Fig. S8a, b, and Fig. S9a, b). Nucleoli-localized SRSF2 protein gradually become displaced from the interior to form foci attached to the surface of shrunken nucleoli (Fig. 4c, Fig. S8a, and Fig. S9a, b). These SRSF2 foci tend to adopt rather irregular morphologies compared to those corresponding to nuclear speckles (Fig. S9a), enabling differentiation of each group of SRSF2 foci in the poly(A)-RNA depleted cells (Fig. S9a–c). During RNase L induced nuclear rearrangements, mCherry probes are expelled from both nuclear speckles and nucleoli (Fig. 4c, d), indicating the reduced accessibility and an increase in the internal packing in both condensates. Consistent with results from probe partitioning, RI imaging revealed an increase in the biomolecular density of nuclear speckles when nuclear RNase L is activated (Fig. 4e–g and Fig. S10a, b). Taken together, using two orthogonal approaches of probing condensate density, RI imaging and probe partitioning, and three different methods of depleting RNA, we found that RNA plays an important role in tuning the biomolecular density of condensates (Fig. 4f, g).

### Intracellular structures influence the distribution, morphology and growth of condensates

Unlike test tubes, living cells are highly crowded with diverse macromolecules and organelles. Utilizing the label-free visualization capability of RI imaging, we sought to probe how crowded intracellular environment modulates the way phase separation proceeds in living cells. For this purpose, we choose to examine stress granules since their assembly can be dynamically induced by applying various stress conditions and they localize to cytoplasm where diverse organelles readily identifiable in RI imaging are present. In U2OS cells, we notice that many cytoplasmic structures with RIs higher than the surrounding cytoplasm are nonuniformly distributed. Using organelle-specific fluorescent probes, we identify that they are mostly mitochondria (Fig. S11). We then use combined fluorescence and RI imaging setup to examine stress granule formation of G3BP1-EGFP expressing cells over time.

**Fig. 5:**
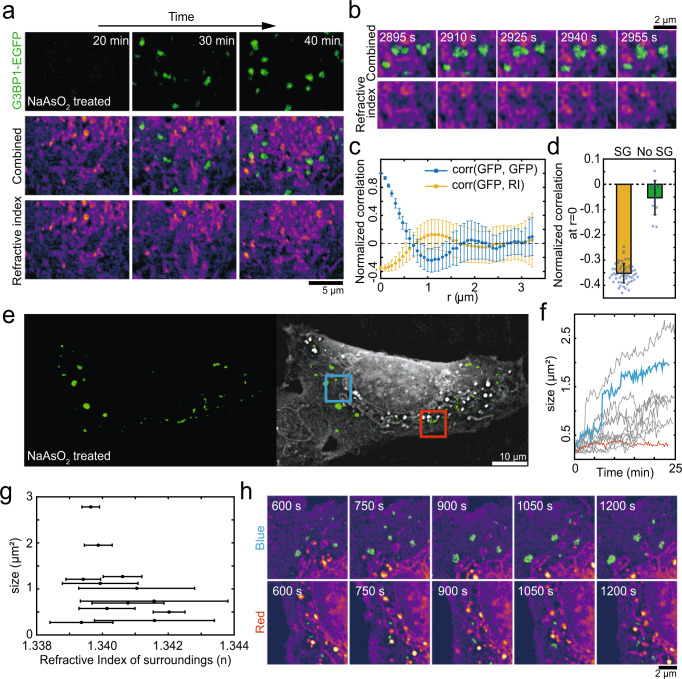
Intracellular structures influence the distribution, morphology and growth of condensates. **a** Time-lapse images of stress granule formation within the G3BP1-EGFP expressing U2OS cell after the treatment of 500 μM sodium arsenite. **b** Zoomed-in images of the same cell in (**a**). **c** Normalized 2D autocorrelation of G3BP1-EGFP images and cross-correlation between G3BP1 and RI images. Plotted are the average (filled circles) and standard deviation (errorbars) of the correlation, at each binned radial shift (*n* = 11). **d** For zero radial shift, normalized cross-correlation values between G3BP1 and RI images are plotted in the presence and absence of stress granules. *n* = 55 (with stress granule) and 11 (no stress granule). Error bar was defined as standard deviation. **e** Combined RI and fluorescence images of a live G3BP1-EGFP expressing U2OS cell one hour after treatment of 500 μM sodium arsenite. Blue and red boxes represent two ROIs in (**h**). **f** Temporal changes of the size of individual stress granules. Granules with color codes are those found in the corresponding ROIs in I. Data from 12 stress granules were shown. **g** The size of individual stress granules at the last time point is plotted against the mean RI values of areas surrounding each granule. RIs within 5 pixels, 385 nm, from each granule are used to compute the mean and the standard deviation (errorbar) of the surroundings. Data from 12 stress granules were shown. **h** Time-lapse images for stress granule growth in two different ROIs. (Top) the blue box ROI in (**e**). (Bottom) the red box ROI in (**e**). All refractive index images are adjusted to 1.337–1.37.

When viewed in the fluorescence channel following sodium arsenite treatment, stress granules appear and grow at seemingly random locations (Fig. 5a). Remarkably, however, when we overlay RI images on top of fluorescence images, we find that stress granules are localized in areas with lower RI values devoid of intracellular structures (Fig. 5a). This indicates that intracellular structures such as mitochondria heavily influence the localization of condensates. Moreover, a closer examination of the condensates reveals that stress granule morphology changes dynamically over time (Fig. 5b). Again, when compared to the corresponding RI images, it is clear that the shape of condensates is also strongly guided by surrounding intracellular structures (Fig. 5b). To quantitatively probe this effect, we perform image correlation analysis for images including those in Fig. 5b (Fig. 5c). The correlation analysis can provide information regarding the size and spatial distribution of structures in images. The radial autocorrelation function of the G3BP1-EGFP images shows a decay over ~ 700 nm, consistent with the overall size of stress granules present in the images. In sharp contrast, the correlation between G3BP1-EGFP fluorescence and corresponding RI images shows negative values for small radial displacements, indicating the presence of a strong anticorrelation between stress granules and intracellular structures with high RI values. When we repeat the same analysis for early time points when stress granules are not yet assembled, no anticorrelation is observed, confirming the validity of our analysis (Fig. 5d).

Typically, stress granules exhibit a broad range of size distribution. We wonder whether intracellular structures can also modulate the growth of condensates. The visual examination of the distribution of intracellular structures and stress granules already suggests that there is likely an anticorrelation between condensate size and the density of nearby intracellular structures (Fig. 5e). To further analyze the origin of size variation, we quantify the growth of individual stress granules over time. We find that there exists a large degree of heterogeneity in the growth rate of each stress granule (Fig. 5f). For individual granules, we compute the average RI values of nearby regions during the time course of stress granule assembly as a metric for the local density of intracellular structures, and then plot it against the size of each granule (Fig. 5g). Indeed, we find that stress granules tend to be smaller in regions with a high density of intracellular structures. A closer inspection of the subregions of cells suggests that intracellular structures can act as a barrier preventing the motion of condensates, which may decrease a chance for fusion-mediated growths (Fig. 5h). Taken together, our data suggest that the crowded intracellular environment can influence the spatial distribution, morphology and growth of intracellular condensates.

## Discussion

In this study, we characterize the total mass concentrations of several condensates within living cells by measuring their refractive index values. We find that intracellular condensates exhibit a broad range of biomolecular densities. The high-density condensates such as nucleoli and heterochromatin exhibit total mass concentrations higher than the surrounding intracellular space. In sharp contrast, under the identical RI imaging conditions, stress granules and nuclear speckles are invisible, indicating that they are low-density structures with their total mass concentrations similar to the surrounding environment. The observed difference in condensate density implies that aqueous solutions take up a higher volume fraction in the internal space of low-density condensates than they do in high-density ones. Indeed, when assayed with fluorescent protein probes, we find that low-density condensates are more permeable, compared to high-density ones. Moreover, consistent with highly porous internal structures, the protein probes in stress granules are exchanged rapidly with cytoplasm as examined in FLIP experiments.

How does the cell define the density of each condensate? In several studies, RNA is shown to be a key factor modulating the phase behavior of many RNA binding proteins^5,65,66^. When we consider an intimate relation between phase diagram and composition, it is likely that RNA is also involved in determining the mass densities of condensates. Indeed, in this study, we find evidence suggesting that RNA plays an important role in regulating the biomolecular density of intracellular condensates. Depleting RNA from nuclear speckles leads to an increase in the total mass concentration, as measured with RI imaging, together with a concomitant exclusion of fluorescence protein probes from speckles. Our results are consistent with previous works showing high enrichment of mRNA in nuclear speckles^54^ and stress granules^55^, and RNA-dependent phase separation of purified condensate components^59,67^.

Then, how does RNA lower the biomolecular density of condensates? In low-density condensates, RNA may act as long scaffolds for recruiting several RNA-binding proteins and form mesh-like transient molecular networks where protein-protein, protein-RNA and RNA-RNA interactions provide intermolecular connectivity^68^. The stretched polymeric nature of RNA can mitigate a requirement for tight physical contacts between proteins that would otherwise increase the density of condensates. Indeed, a mixture of purified G3BP1 and RNA forms in-vitro liquid droplets with the condensed phase G3BP1 concentration of only 1 mg/ml (~15 µM)^59^, about a few hundred times lower than protein-only condensates^25,26^. Previously reported heterogeneous internal organization of stress granules and nuclear speckles, exhibiting denser cores surrounded by shells^11,58,69,70^, is also consistent with mesh-like internal architecture. Notably, we found that RNA depletion leads to stronger exclusion of protein probes not just from low-density condensates such as nuclear speckles, but also from nucleoli (Fig. 4c, d). Thus, cellular RNA appears to regulate the biomolecular density of condensates in general. Then, unlike nuclear speckles, why does nucleolus, containing a site of rRNA production, exhibit the compact internal organization with high density? In nucleoli, nascent rRNA transcripts undergo directional flux towards the outside of the condensate while progressively assembling with multiple ribosomal proteins to form pre-ribosomal particles^71^. The presence of secondary structures and/or specific binding configurations of rRNA with ribosomal proteins may influence the phase behaviors of nucleoli in a way to increase its density. Consistent with this view, unlike mRNA, rRNA cannot promote in-vitro phase separation of several RNA binding proteins, including G3BP1 unless it is unfolded^59,72^. In addition, a recent work showed that partitioning of pre-rRNA into in-vitro droplets of nucleolar component fibrillarin requires correctly folded rRNA^73^. From a functional point of view, the densely-packed internal environment of nucleoli can facilitate thermodynamic exclusion of fully assembled pre-ribosome subunits from nucleoli^74^.

**Fig. 6:**
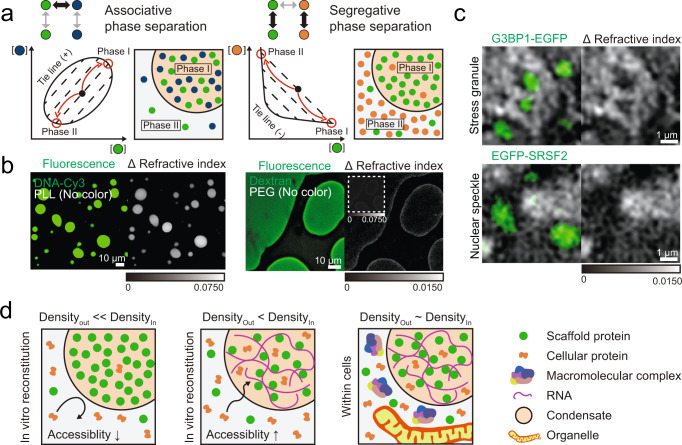
Demixing transition of low-density condensates in living cells. **a** Schematics of phase diagrams for associative- (left) and segregative phase separation (right). In the simple ternary systems, different intermolecular interactions can give rise to immiscible phases of distinct compositions, which can be represented in terms of phase diagrams. The ternary mixtures with initial compositions labeled as black dots undergo phase separation along the tie line to form two immiscible phases. **b** (Left) Representative fluorescence and ΔRI images of the mixture of DNA and poly-l-lysine (PLL). The mixture contains 89.5 μM DNA phosphates (10% FAM labeled) and 0.068 w/v% PLL amines in 100 mM NaCl buffer. (Right) Representative fluorescence and ΔRI images of PEG-dextran aqueous two-phase system (ATPS). 5 wt% TMR-labeled dextran and 4 wt% PEG are used in the ATPS system. The inset image is adjusted to the range of ΔRI 0–0.075 for comparison with the DNA-PLL image. ΔRI images are obtained by subtracting the minimum RI pixel value of each raw RI image. **c** (Top) Combined RI and fluorescence images of stress granules in U2OS cells expressing G3BP1-EGFP. (Bottom) Combined ΔRI and fluorescence images of nuclear speckles in U2OS cells expressing EGFP-SRSF2. For comparison, the scale of RIs is adjusted to be identical to that in (**b**, right). **d** Schematic illustration of the demixing transition model of low-density condensates. RNA-mediated associative phase separation can lead to the assembly of condensates with reduced biomolecular density, compared to protein-only ones. An increase in the available internal space facilitated by RNA promotes the access of cellular proteins into condensates. Within living cells, diverse macromolecular complexes as well as organelles may segregatively work to further enhance phase separation of condensate components. Together, the content of living cells undergoes the demixing transition to form low-density condensates where a minimal density difference is observed across condensate boundary.

Segregative phase separation may act as another mechanism, not mutually exclusive with the RNA-mediated phase separation described above, for the formation of low-density condensates. Depending on types of intermolecular interactions, phase separation of aqueous polymer solutions can be largely categorized into two different classes: associative- and segregative phase separation^75–78^. Driven by attractive intermolecular interactions, associative phase separation leads to the formation of a polymer-rich phase and the other phase deprived of polymers. Examples include phase separations of self-associating proteins such as prion-like proteins and RNA binding proteins^1,5,66,79^, as well as complex coacervation^19^ between oppositely charged macromolecules and phase separations of multivalent protein domains^13,80^. In terms of density, these examples can be thought of as a condensation process, giving rise to a dense phase with high polymer density surrounded by a dilute phase of low density. In contrast, segregative phase separation involves non-associative or repulsive interactions between polymers. A classic example includes the aqueous two-phase system of PEG and dextran^75,76^. In segregative phase separation, neither of phases predominantly take up high concentrations of polymers: two polymers demix into distinct phases, each of which is comprised mostly of one type of the polymers. Thus, segregative phase separation can lead to the formation of two phases with similar densities. To demonstrate this situation, we prepare a mixture of TMR-labeled dextran and PEG (Fig. 6a). Consistent with previous works^75,81^, we observe strong segregative liquid-liquid phase separation of the mixture, with dye-labeled dextran highly enriched in one of the phases. When imaged using our ODT setup, we find that a difference in the RI between the dextran-rich phase and the PEG-rich phase is indeed minimal (Fig. 6b), similar to what we find between low-density condensates and their surroundings (Fig. 6c). In sharp contrast, associative phase separation of DNA and poly-l-lysine gives rise to two coexisting phases, the densities of which yet differ greatly with one another (Fig. 6b). We speculate that diverse macromolecule species and organelles present at highly crowded level in the cell can contribute to segregative phase separation. Indeed, we show that intracellular structures such as mitochondria heavily influence the localization and dynamic growth of stress granules (Fig. 5).

Our results illustrate that, rather than simply a site where various biomolecules are locally concentrated, intracellular condensates display a spectrum of biomolecular densities, some even exhibiting densities similar to the surrounding protoplasm. We postulate that in living cells, the demixing transition involving the segregative mechanism together with RNA-mediated selective associations collectively gives rise to low-density condensates (Fig. 6d). Other mechanisms such as criticality may further contribute to the behaviors of intracellular phases^82^, which are exciting topics to be probed in future studies. The density of condensates likely has a strong influence on the way biomolecules behave in cellular milieu, and thus may play an important role for bringing about functional outcomes. Future work will explore the functional consequences of regulating condensate density in the crowded intracellular environment.

## Methods

### Cell culture

Lenti-X 293 T (Takara, 632180) cells and NIH3T3 (KCLB, 21658) cells were cultured in 10% FBS (HyClone, SV30207.02) DMEM (Sigma-Aldrich, D6429) supplemented with penicillin/streptomycin antibiotics (Gibco, 15140122) at 37 °C with 5% CO_2_ in a humidified incubator. U2OS (KCLB, 30096) cells were grown in the presence of GlutaMAX (Gibco, 35050061).

### Plasmid construction

To generate fluorescent protein tagged condensate markers, cDNA clones encoding human NPM1, human SRSF2, human SRSF1, human G3BP1, human HP1α, human FIB, and human Sec61B were obtained from Korea Human Gene Bank (Medical Genomics Research Center, Korea). From each cDNA, DNA fragments were amplified by PCR using HiFi PCR premix (Takara, 639298). To generate NLS-RNase L-P2A-BFP, the gBlock gene fragment encoding NLS-RNase L was obtained from Integrated DNA Technologies. Each fragment was assembled into the pHR lentiviral backbone using In-Fusion Cloning Kit (Takara, 639649). The resulting constructs were fully sequenced to confirm the absence of unwanted substitutions.

### Antibodies

Primary antibodies used in this study were mouse monoclonal anti-SRSF2 (Abcam, ab11826, 1:200 dilution), mouse monoclonal anti-NPM1 (Santi Cruz, sc-56622, 1:100 dilution), mouse monoclonal anti-G3BP1 (Abcam, ab56574, 1:200 dilution), and rabbit monoclonal anti-HP1α (Abcam, ab109028, 1:250 dilution). Secondary antibodies specific to rabbit IgG conjugated with Alexa 488 (Invitrogen, A-11008, 1:500 dilution) and mouse IgG conjugated with Alexa 546 (Invitrogen, A-11030, 1:500 dilution) were used.

### Immunocytochemistry experiments

Cells were plated on Tomodish (Tomocube) with 30% confluency one day before fixation. For immunocytochemistry, cells were fixed with 4% formaldehyde solution (Invitrogen, FB002) for 10 min and washed once with 0.5% PBS-T (PBS supplemented with 0.5% Triton X-100, Cayman chemical company, A35316). After incubation in 0.5% PBS-T for 5 min, dishes were rinsed with 0.1% PBS-T (0.1% Triton X-100 in PBS). After 7 minutes of incubation in 0.1% PBS-T, the cells were blocked with blocking solution (0.1% PBS-T and 10% fetal bovine serum, HyClone, SV30207.02) for 30 min, followed by incubation with primary antibodies diluted in blocking solution for 1 hour, washing 5 times every 7 minutes with 0.1% PBS-T, incubation with secondary antibodies in blocking solution for 1 hour and washing 5 times every 7 min with 0.1% PBS-T.

### RNA FISH

Cells were seeded on confocal dish (SPL, 100350) at 30-50% confluency one day before fixation. For poly-dT RNA FISH, cells were fixed with 4% formaldehyde solution (Invitrogen, FB002) for 10 min and washed once with 0.5% PBS-T (PBS supplemented with 0.5% Triton X-100, Cayman chemical company, A35316). After incubation in 0.5% PBS-T for 5 min, dishes were rinsed with 0.1% PBS-T (0.1% Triton X-100 in PBS), and incubated in 0.1% PBS-T for 5 min. Then, the cells were washed 2 times with PBS, followed by 5 min incubation in 1 ml Wash buffer (10% SSC, Invitrogen, AM9763 and 10% formamide, Invitrogen, 15515026 in UPW). The samples were further washed with 1 ml PBS once, and incubated in 42 °C incubator for 4–16 h in the mixture of 1 ml PBS and 35 μl hybridization buffer (50% dextran sulfate, Cayman chemical company, A44635, 10% formamide, 10% SSC, and 150 nM 30-mer poly-dT FISH probe dissolved in ultra-pure water). One hour before imaging, the samples were shaken on a tabletop shaker in the dark for 30 min, then the media was replaced with 1 ml Wash buffer and incubated for 5 min. After washing with 1 ml PBS twice, imaging was performed.

### Cell transfection

For imaging, cells were transfected using Lipofectamine 3000 transfection reagent (Invitrogen, L3000015) following the manufacturer’s instructions. For lentivirus production, FuGene HD (Promega, E2311) was used alternatively.

### Cell line construction

For Lenti-X 293 T cells plated on a 6-well plate 1 day prior to transfection, pHR vectors containing desired DNA constructs (1.5 µg), pCMVdR8.91 (1.33 µg), and pMD2.G (0.17 µg) were cotransfected using FuGene HD (Promega, E2311) according to manufacturer’s instructions. Within 2–3 days after transfection, 2 mL of viral supernatants was collected and filtered to remove cell debris using 0.45 µm filter (Millipore, SLHVR033RS). For NIH3T3 and U2OS cells at 30% confluency on 60 mm tissue culture dishes, cells were transduced by dropping each of lentivirus-containing solutions to the cell medium with a designed amount suitable for desirable expression levels.

### Stress treatment

Oxidative stress to U2OS and NIH3T3 cells was induced by treating cells with 250–500 μM sodium arsenite (Sigma-Aldrich, S7400) in regular cell culture conditions.

### RNA depletion experiment

For transcription inhibition, 5,6-dichloro-benzimidazole riboside (DRB; Sigma-Aldrich, D1916) and Actinomycin-D (ActD; Sigma-Aldrich, A9415) were purchased from Merck. DRB and ActD were dissolved in dimethyl sulfoxide (DMSO; Sigma-Aldrich, D2650) to be a concentration of 10 mM or 500 µg/ml, respectively. One day before imaging, cells were plated on confocal dish (SPL, 100350) or Tomodish (Tomocube) at 30% confluency. Cells were treated with DRB or ActD at a final concentration of 100 µM or 5 µg/ml, respectively. Imaging of drug-treated cells was performed up to 6 hours after DRB treatment or 12 hours after ActD treatment.

For NLS-RNase L activation, poly(I:C) HMW (InvivoGen: tlrl-pic) was dissolved in the company-provided endotoxin-free physiological water to obtain a solution of 1 mg/ml. One day before imaging, cells stably expressing NLS-RNase L-P2A-BFP were plated on confocal dish (SPL, 100350) or Tomodish (Tomocube) at 50% confluency. Cells were transfected with 2.5 µg poly(I:C) using 9 µl of FuGene HD (Promega, E2311) or mock transfected using FuGene alone. Imaging was performed up to 5 hours after the transfection.

### MitoTracker staining

For mitochondrial staining, MitoTracker Red FM (MT; Invitrogen, M22425) was purchased from ThermoFisher. MT was dissolved in dimethyl sulfoxide (DMSO; Sigma-Aldrich, D2650) to a final concentration of 1 mM and stored at −20 °C. One day before imaging, cells were plated on Tomodish (Tomocube) at 30% confluency. On the day of imaging, the growth media was exchanged with prewarmed staining solution containing MT probe at a final concentration 300–400 nM. After 30-minute incubation, the staining media was replaced with fresh prewarmed media and imaging was performed immediately.

### Live cell imaging (Confocal microscopy)

Cells were plated on the TC treated confocal dish (SPL, 100350) and grown in regular growth medium to reach ~60% confluency. Live cell imaging was performed using a Nikon 60X oil immersion objective (NA 1.4; Nikon, MXA22168) on a Nikon A1 laser scanning confocal microscope equipped with a CO_2_ microscope stage incubator under 5% CO_2_ and 37°C.

### Fluorescence loss in photobleaching (FLIP)

NIH3T3 expressing G3BP1-mCh, EGFP, and miRFP was plated on the TC treated confocal dish 1 day before imaging. Nikon A1 confocal microscope with Nikon 60X oil immersion objective (NA 1.4; Nikon, MXA22168) was used to perform FLIP experiments. To induce stress granule formation, cells were treated with sodium arsenite (Sigma-Aldrich, S7400) at the final concentration of 250 μM. The location of bleaching area as well as illumination conditions were chosen so that bleaching lasers did not cause direct changes in fluorescence signals for the stress granule being monitored for probe mobility. After taking 5 initial images, total 100 cycles of bleaching and imaging were performed. A single cycle is comprised of bleaching for 488 nm, 561 nm, and 640 nm channels, followed by acquisition of 15 images every 3 s. To create fluorescence recovery curves, fluorescence intensities were normalized with the average intensities of initial 5 images.

### Optical diffraction tomography

A commercial ODT system (HT-2H, Tomocube Inc., Republic of Korea) was used to measure the 3D RI tomograms of individual cells and subcellular condensates. The optical setup is based on Mach-Zehnder interferometry. A laser beam from a diode-pumped solid-state laser (532 nm) was split into two beam ways: a sample beam and a reference beam path. A digital micromirror device, located at the conjugate plane of the sample, allows for rapid control of incident angles of the illumination plane waves by displaying the grating patterns at desired periods and directions^43^. The plane wave at the controlled angle illuminated a sample via a high numerical aperture (NA) objective lens (NA = 1.2, water immersion, UPLSAPO 60XW, Olympus Inc.), and the diffracted wave from the sample was collected via the high NA objective lens (NA = 1.2, water immersion, UPLSAPO 60XW, Olympus Inc.). The sample and the reference beam combined through a beam splitter and generate spatially modulated holograms in a camera plane, which were recorded using a CMOS camera. The total acquisition time for 50 holograms was approximately 0.1 s. From the 2D holograms acquired with various illumination angles, the optical field images, containing both the amplitude and phase images, were retrieved using a field retrieval algorithm^83^. Then, each optical field image was mapped onto an Ewald surface in 3D Fourier space, using the principle of ODT^44^. Due to the limited NAs of both the condenser and objective lenses, side scattering signals were not collected, resulting into the deterioration of axial resolution, which is known as a missing cone problem. To resolve the missing cone problem, an iterative regularization algorithm with the non-negativity constraint was used^84^. The detailed principle and algorithm on ODT can be found elsewhere^85^. Unless specifically mentioned, presented RI images are individual slices from 3D RI tomograms and all RI quantifications were based on 3D RI distributions. The theoretical lateral and axial resolution of the used ODT system were 110 nm and 360 nm, respectively. The precision of the ODT was characterized by measuring the RIs of identical 1.6 µm silica beads (Thermofisher, 8150; RI = 1.46) 20 times and then repeating this procedure for multiple beads to compute the averaged standard error, yielding 4.8 × 10^−4^. For live cell RI and fluorescence imaging, cells were plated on Tomodish (Tomocube) one day before imaging at 20% confluency in regular growth media supplemented with GlutaMax (Gibco, 35050061). Prior to imaging, the media was replaced with 37 °C pre-warmed fresh media to wash out cell debris. The microscope was equipped with a chamber maintaining 5% CO_2_ and 37 °C.

### PEG/Dextran phase separation experiments

Polyethylen glycol (PEG) and dextran were used to form an aqueous two-phase system. Tetramethylrhodamine isothiocyanate-dextran (Sigma-Aldrich, T1287; average Mw 155,000 Da) and PEG (BioUltra, 89510; Mw 7000–9000 Da) were purchased from Merck. PEG and dextran were dissolved in ultrapure waters to be stocked as 10 wt% solutions. For desired concentrations, an adequate amount of PEG and dextran stock were mixed with ultrapure water. A difference of RIs between PEG-rich phase and dextran-rich phase was measured using HT-2H.

### ssDNA/poly-l-lysine (PLL) phase separation experiments

ssDNA sequence: 5’- CCTTCCCTCCACCCCACCCTCCCCTCCC – 3’.

The DNA oligonucleotide (Mw 8237) was purchased from Bionics, and the powder form of PLL-hydrobromide (Sigma-Aldrich, P1274; Mw 70,000 – 150,000) from Merck. The molecular weights of the materials were chosen to be as similar as possible to the pair of PEG and dextran. Lyophilized DNA oligo and PLL were dissolved in ultrapure water to make stocks for storage. In an effort to maximize the volume fraction of the dense-droplet phase, samples were mixed under the condition of [amines]:[phosphates]=1:1. The final sample is a mixture of 89.5 μM DNA oligo (10% labeled with FAM dye) and 0.068 w/v% PLL in a buffer of 100 mM NaCl and 20 mM Tris-HCl. A difference of RIs between two different phases was measured using HT-2H.

### Estimation of refractive index increments for condensate proteome

All protein sequence data as well as a list of entire human proteome were obtained from UniprotKB/Swiss-Prot, and lists of protein components for nucleoli^39^, heterochromatin^41^, nuclear speckles^39^ and stress granules^40^ were obtained from previous studies. The detailed procedure of predicting the distribution of refractive index increments for each proteome based on the sequence of individual proteins is described elsewhere^38^. All analysis was performed using custom-built MATLAB scripts.

### Data analysis

To quantify the refractive indices of nucleoli and heterochromatin, we used two different types of cell images, the 3D RI tomograms of intact live cells and immunofluorescence images of the same cells after fixation. Immunofluorescence images were used to identify locations of individual condensates. Due to time differences inherently present in these two datasets, not all condensates were identifiable in live-cell RI images. Since the ODT experiences a resolution limit from diffraction, only those condensates larger than around 0.44 µm in diameter were included in our analysis of the RI measurements. In addition, heterochromatin regions positioned near or inside of nucleoli were excluded from RI quantifications. The total mass concentration was estimated using measured refractive indices and the RI increment for protein and nucleic acids (dn/dc = 0.19 mL/g)^38^. In this study, the terms, total mass concentration and mass density, are used interchangeably. To quantify the refractive indices of stress granules and nuclear speckles, fluorescent protein-tagged marker proteins, such as EGFP-SRSF2 for nuclear speckles and G3BP1-EGFP for stress granules, were expressed in each cell type for identification of individual condensates. Only condensates larger than approximately 0.44 µm in diameter were analyzed for RI measurements to prevent any unwanted underestimation associated with the resolution limit. In RNA depletion conditions, some SRSF2 proteins tend to relocate to the nucleoli. As detailed in Fig. S9, SRSF2-positive foci were classified into nucleolus- and speckle-associated ones. Only those classified into speckle-associated ones were included in our analysis of probe partitioning, poly-dT intensity measurement, and RI measurements. For mock or DMSO-treated samples, only size thresholding is applied. The line intensity profiles as well as kymographs were generated using ImageJ. The image correlation analysis was performed using custom-built MATLAB scripts.

### Statistics and Reproducibility

The experimental data are represented as the mean ± std in all boxplots except for Fig. 1e, S7f, and S7f, where the center line is the mean, box limits are Q1 and Q3, and whiskers are Max and Min. All boxplots were generated using custom-built MATLAB scripts. Two-sided unpaired t*-*test was used to calculate the statistical significance of differences between two experimental groups and P-values less than 0.05 were considered significant. All statistical analysis was performed using custom-built MATLAB scripts. All microscopic, biochemical, and biological assays were independently repeated at least three times.

### Reporting summary

Further information on research design is available in the Nature Portfolio Reporting Summary linked to this article.

## Supplementary information

Supplementary Information

Description of Additional Supplementary Files

Supplementary Movie 1

Supplementary Movie 2

Reporting Summary

## Data Availability

All relevant data supporting the key findings of this study are available within the article or from the corresponding author upon request.

## Code availability

Custom MATLAB code used in this study is available upon request.

## References

[1] Banani SF, Lee HO, Hyman AA, Rosen MK (2017). Biomolecular condensates: Organizers of cellular biochemistry. Nat. Rev. Mol. Cell Biol..

[2] Shin Y, Brangwynne CP (2017). Liquid phase condensation in cell physiology and disease. Science.

[3] Brangwynne CP (2009). Germline P granules are liquid droplets that localize by controlled dissolution/condensation. Science.

[4] Feric M (2016). Coexisting liquid phases underlie nucleolar subcompartments. Cell.

[5] Molliex A (2015). Phase separation by low complexity domains promotes stress granule assembly and drives pathological fibrillization. Cell.

[6] Marzahn, M. R. et al. Higher-order oligomerization promotes localization of SPOP to liquid nuclear speckles. *EMBO J.* **35**, 1254–1275 (2016). 10.15252/embj.201593169 PMC4910529 27220849

[7] Strom AR (2017). Phase separation drives heterochromatin domain formation. Nature.

[8] Woodruff JB (2017). The centrosome is a selective condensate that nucleates microtubules by concentrating tubulin. Cell.

[9] Case LB, Zhang X, Ditlev JA, Rosen MK (2019). Stoichiometry controls activity of phase-separated clusters of actin signaling proteins. Science.

[10] Brangwynne C, Mitchison T, Hyman AA (2011). Active liquid-like behavior of nucleoli determines their size and shape in Xenopus laevis oocytes. Proc. Natl. Acad. Sci. USA.

[11] Wheeler JR, Matheny T, Jain S, Abrisch R, Parker R (2016). Distinct stages in stress granule assembly and disassembly. Elife.

[12] Frottin F (2019). The nucleolus functions as a phase-separated protein quality control compartment. Science.

[13] Li P (2012). Phase transitions in the assembly of multivalent signalling proteins. Nature.

[14] Milovanovic D, Wu Y, Bian X, De Camilli P (2018). A liquid phase of synapsin and lipid vesicles. Science.

[15] Dao TP (2018). Ubiquitin Modulates Liquid-Liquid Phase Separation of UBQLN2 via Disruption of Multivalent Interactions. Mol. Cell.

[16] Sabari BR (2018). Coactivator condensation at super-enhancers links phase separation and gene control. Science.

[17] Zeng M (2016). Phase transition in postsynaptic densities underlies formation of synaptic complexes and synaptic plasticity. Cell.

[18] Harmon TS, Holehouse AS, Rosen MK, Pappu RV, States U (2017). Intrinsically disordered linkers determine the interplay between phase separation and gelation in multivalent proteins. Elife.

[19] Pak CW (2016). Sequence determinants of intracellular phase separation by complex coacervation of a disordered protein. Mol. Cell.

[20] Wang J (2018). A molecular grammar governing the driving forces for phase separation of prion-like RNA binding proteins. Cell.

[21] Nott TJ (2015). Phase transition of a disordered nuage protein generates environmentally responsive membraneless organelles. Mol. Cell.

[22] Martin EW (2020). Valence and patterning of aromatic residues determine the phase behavior of prion-like domains. Science.

[23] Espinosa JR (2020). Liquid network connectivity regulates the stability and composition of biomolecular condensates with many components. Proc. Natl. Acad. Sci. USA.

[24] Sanders DW (2020). Competing protein-RNA interaction networks control multiphase intracellular organization. Cell.

[25] Burke KA, Janke AM, Rhine CL, Fawzi NL (2015). Residue-by-residue view of in vitro FUS granules that bind the C-terminal domain of RNA Polymerase II. Mol. Cell.

[26] Brady JP (2017). Structural and hydrodynamic properties of an intrinsically disordered region of a germ cell-specific protein on phase separation. Proc. Natl. Acad. Sci. USA.

[27] Alberti S (2018). A User’s guide for phase separation assays with purified proteins. J. Mol. Biol..

[28] Milo R (2013). What is the total number of protein molecules per cell volume? A call to rethink some published values. BioEssays.

[29] Jacobs WM, Frenkel D (2017). Phase transitions in biological systems with many components. Biophys. J..

[30] Barer R, Tkaczyk S (1954). Refractive index of concentrated protein solutions. Nature.

[31] Zangle TA, Teitell MA (2014). Live-cell mass profiling: An emerging approach in quantitative biophysics. Nat. Methods.

[32] Barer R, Ross KFA, Tkaczyk S (1953). Refractometry of living cells. Nature.

[33] Tumolo T, Angnes L, Baptista MS (2004). Determination of the refractive index increment (dn/dc) of molecule and macromolecule solutions by surface plasmon resonance. Anal. Biochem..

[34] Di Primo C, Lebars I (2007). Determination of refractive index increment ratios for protein-nucleic acid complexes by surface plasmon resonance. Anal. Biochem..

[35] Barer R (1952). Interference microscopy and mass determination. Nature.

[36] Schürmann M, Scholze J, Müller P, Guck J, Chan CJ (2016). Cell nuclei have lower refractive index and mass density than cytoplasm. J. Biophotonics.

[37] McCall, P. M. et al. Quantitative phase microscopy enables precise and efficient determination of biomolecular condensate composition. *bioRxiv*10.1101/2020.10.25.352823 (2020).

[38] Zhao H, Brown PH, Schuck P (2011). On the distribution of protein refractive index increments. Biophys. J..

[39] Thul PJ (2017). A subcellular map of the human proteome. Science.

[40] Youn JY (2019). Properties of stress granule and P-Body proteomes. Mol. Cell.

[41] Becker JS (2017). Genomic and proteomic resolution of heterochromatin and its restriction of alternate fate genes. Mol. Cell.

[42] Park YK, Depeursinge C, Popescu G (2018). Quantitative phase imaging in biomedicine. Nat. Photonics.

[43] Shin S, Kim K, Yoon J, Park Y (2015). Active illumination using a digital micromirror device for quantitative phase imaging. Opt. Lett..

[44] Wolf E (1969). Three-dimensional structure determination of semi-transparent objects from holographic data. Opt. Commun..

[45] Sandoz PA, Tremblay C, van der Goot FG, Frechin M (2019). Image-based analysis of living mammalian cells using label-free 3D refractive index maps reveals new organelle dynamics and dry mass flux. PLoS Biol..

[46] Kim K, Guck J (2020). The relative densities of cytoplasm and nuclear compartments are robust against strong perturbation. Biophys. J..

[47] Choi W (2007). Tomographic phase microscopy. Nat. Methods.

[48] Larson AG (2017). Liquid droplet formation by HP1α suggests a role for phase separation in heterochromatin. Nature.

[49] Imai R (2017). Density imaging of heterochromatin in live cells using orientation-independent-DIC microscopy. Mol. Biol. Cell.

[50] Lamond AI, Spector DL (2003). Nuclear speckles: A model for nuclear organelles. Nat. Rev. Mol. Cell Biol..

[51] Kedersha N, Ivanov P, Anderson P (2013). Stress granules and cell signaling: More than just a passing phase?. Trends Biochem. Sci..

[52] Bancaud A (2009). Molecular crowding affects diffusion and binding of nuclear proteins in heterochromatin and reveals the fractal organization of chromatin. EMBO J..

[53] Schellenberg B (2013). Bax exists in a dynamic equilibrium between the cytosol and mitochondria to control apoptotic priming. Mol. Cell.

[54] Hall LL, Smith KP, Byron M, Lawrence JB (2006). Molecular anatomy of a speckle. Anat. Rec. - Part A Discov. Mol. Cell. Evol. Biol..

[55] Kedersha NL, Gupta M, Li W, Miller I, Anderson P (1999). RNA-binding proteins TIA-1 and TIAR link the phosphorylation of eIF-2α to the assembly of mammalian stress granules. J. Cell Biol..

[56] Shevtsov SP, Dundr M (2011). Nucleation of nuclear bodies by RNA. Nat. Cell Biol..

[57] Bounedjah O (2014). Free mRNA in excess upon polysome dissociation is a scaffold for protein multimerization to form stress granules. Nucleic Acids Res..

[58] Fei J (2017). Quantitative analysis of multilayer organization of proteins and RNA in nuclear speckles at super resolution. J. Cell Sci..

[59] Guillén-Boixet J (2020). RNA-induced conformational switching and clustering of G3BP drive stress granule assembly by condensation. Cell.

[60] Steurer B (2018). Live-cell analysis of endogenous GFP-RPB1 uncovers rapid turnover of initiating and promoter-paused RNA Polymerase II. Proc. Natl. Acad. Sci. USA.

[61] Sobell HM (1985). Actinomycin and DNA transcription. Proc. Natl. Acad. Sci. USA.

[62] Bregman DB, Du L, Van Der Zee S, Warren SL (1995). Transcription-dependent redistribution of the large subunit of RNA polymerase II to discrete nuclear domains. J. Cell Biol..

[63] Decker CJ, Burke JM, Mulvaney PK, Parker R (2022). RNA is required for the integrity of multiple nuclear and cytoplasmic membrane‐less RNP granules. EMBO J.

[64] Burke JM, Moon SL, Matheny T, Parker R (2019). RNase L reprograms translation by widespread mRNA turnover escaped by antiviral mRNAs. Mol. Cell.

[65] Lin Y, Protter DSW, Rosen MK, Parker R (2015). Formation and maturation of phase-separated liquid droplets by RNA-binding proteins. Mol. Cell.

[66] Maharana S (2018). RNA buffers the phase separation behavior of prion-like RNA binding proteins. Science.

[67] Yang P (2020). G3BP1 is a tunable switch that triggers phase separation to assemble stress granules. Cell.

[68] Tauber D, Tauber G, Parker R (2020). Mechanisms and Regulation of RNA Condensation in RNP Granule Formation. Trends Biochem. Sci..

[69] Jain S (2016). ATPase-modulated stress granules contain a diverse proteome and substructure. Cell.

[70] Niewidok B (2018). Single-molecule imaging reveals dynamic biphasic partition of RNA-binding proteins in stress granules. J. Cell Biol..

[71] Lafontaine DLJ, Riback JA, Bascetin R, Brangwynne CP (2021). The nucleolus as a multiphase liquid condensate. Nat. Rev. Mol. Cell Biol..

[72] Saha S (2016). Polar positioning of phase-separated liquid compartments in cells regulated by an mRNA competition mechanism. Cell.

[73] Yao RW (2019). Nascent Pre-rRNA sorting via phase separation drives the assembly of dense fibrillar components in the human nucleolus. Mol. Cell.

[74] Riback JA (2020). Composition-dependent thermodynamics of intracellular phase separation. Nature.

[75] Aumiller WM, Keating CD (2017). Experimental models for dynamic compartmentalization of biomolecules in liquid organelles: Reversible formation and partitioning in aqueous biphasic systems. Adv. Colloid. Interface Sci..

[76] Ribeiro SS, Samanta N, Ebbinghaus S, Marcos JC (2019). The synergic effect of water and biomolecules in intracellular phase separation. Nat. Rev. Chem..

[77] André AAM, Spruijt E (2020). Liquid–liquid phase separation in crowded environments. Int. J. Mol. Sci..

[78] Shin Y (2022). Rich phase separation behavior of biomolecules. Mol. Cells.

[79] Alberti S, Gladfelter A, Mittag T (2019). Considerations and challenges in studying liquid-liquid phase separation and biomolecular condensates. Cell.

[80] Banani SF (2016). Compositional control of phase-separated cellular bodies. Cell.

[81] Haynes CA, Beynon RA, King RS, Blanch HW, Prausnitz JM (1989). Thermodynamic properties of aqueous polymer solutions: Poly(ethylene glycol)/dextran. J. Phys. Chem..

[82] Veatch SL (2008). Critical fluctuations in plasma membrane vesicles. ACS Chem. Biol..

[83] Debnath SK, Park Y (2011). Real-time quantitative phase imaging with a spatial phase-shifting algorithm. Opt. Lett..

[84] Lim J (2015). Comparative study of iterative reconstruction algorithms for missing cone problems in optical diffraction tomography. Opt. Express.

[85] Kim K (2013). High-resolution three-dimensional imaging of red blood cells parasitized by Plasmodium falciparum and in situ hemozoin crystals using optical diffraction tomography. J. Biomed. Opt..

