# Supplementary material for: RNA-mediated demixing transition of low-density condensates
Source: Nat Commun. 2023 Apr 27;14:2425. doi: 10.1038/s41467-023-38118-z (PMC10140143; doi:10.1038/s41467-023-38118-z)
Supplement: Supplementary file 1 — Supplementary Information [file 41467_2023_38118_MOESM1_ESM.pdf]

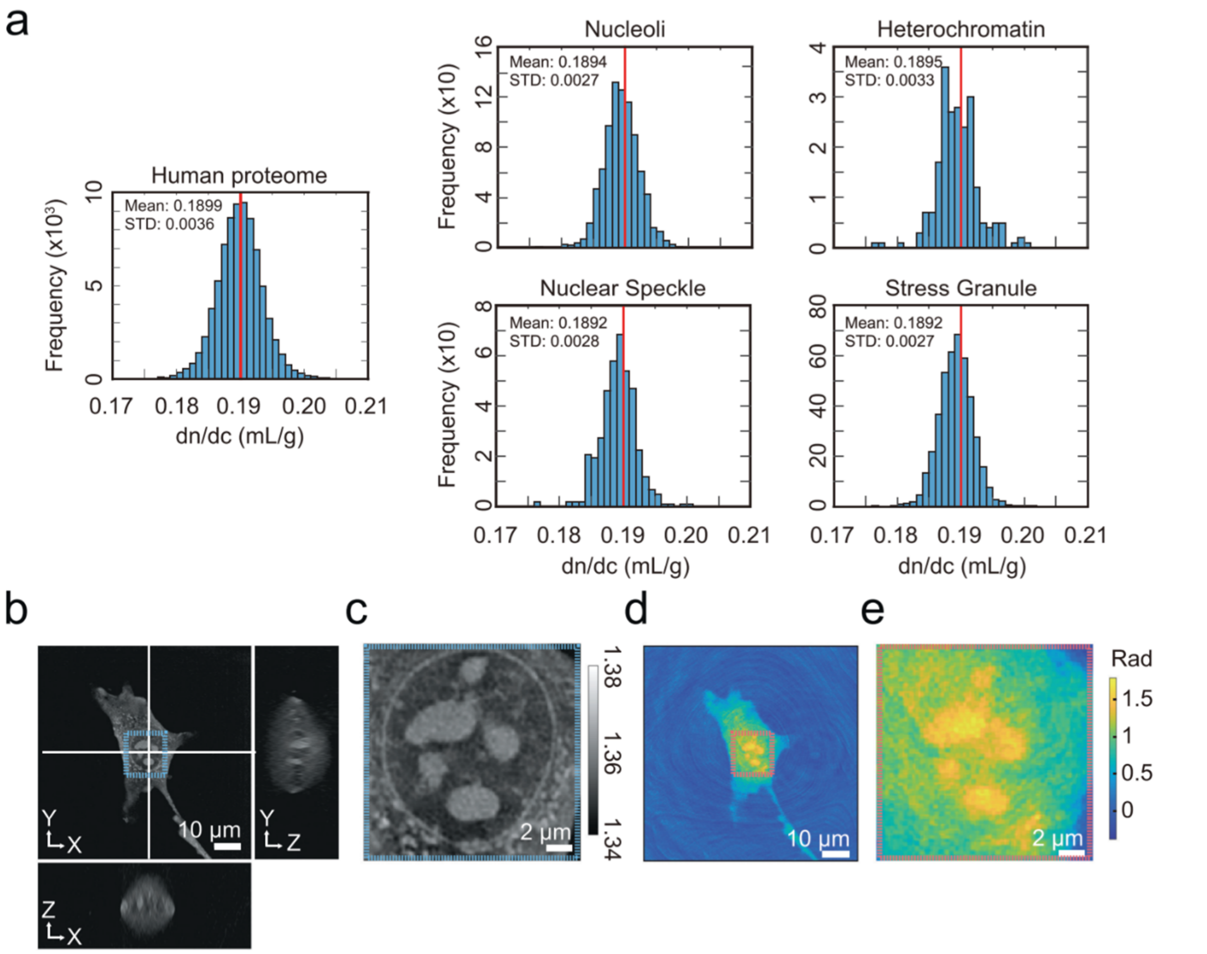

Fig S1. Refractive index increment distributions and comparison between 2D QPI and ODT.

a Histograms of the estimated refractive index increments of individual proteins for the entire human proteome and four condensate types used in this study (see Methods for the detailed procedure). Red lines indicate 0.19 ml/g. **b** Cross-sectional view of the 3D RI tomograms of the NIH-3T3 cell used in the Fig. 1d. **c** The zoomed-in image of the dashed area in (**b**). **d** 2D QPI image of the same cell in (b-c). **e** The zoomed-in image of the dashed area in (**d**).

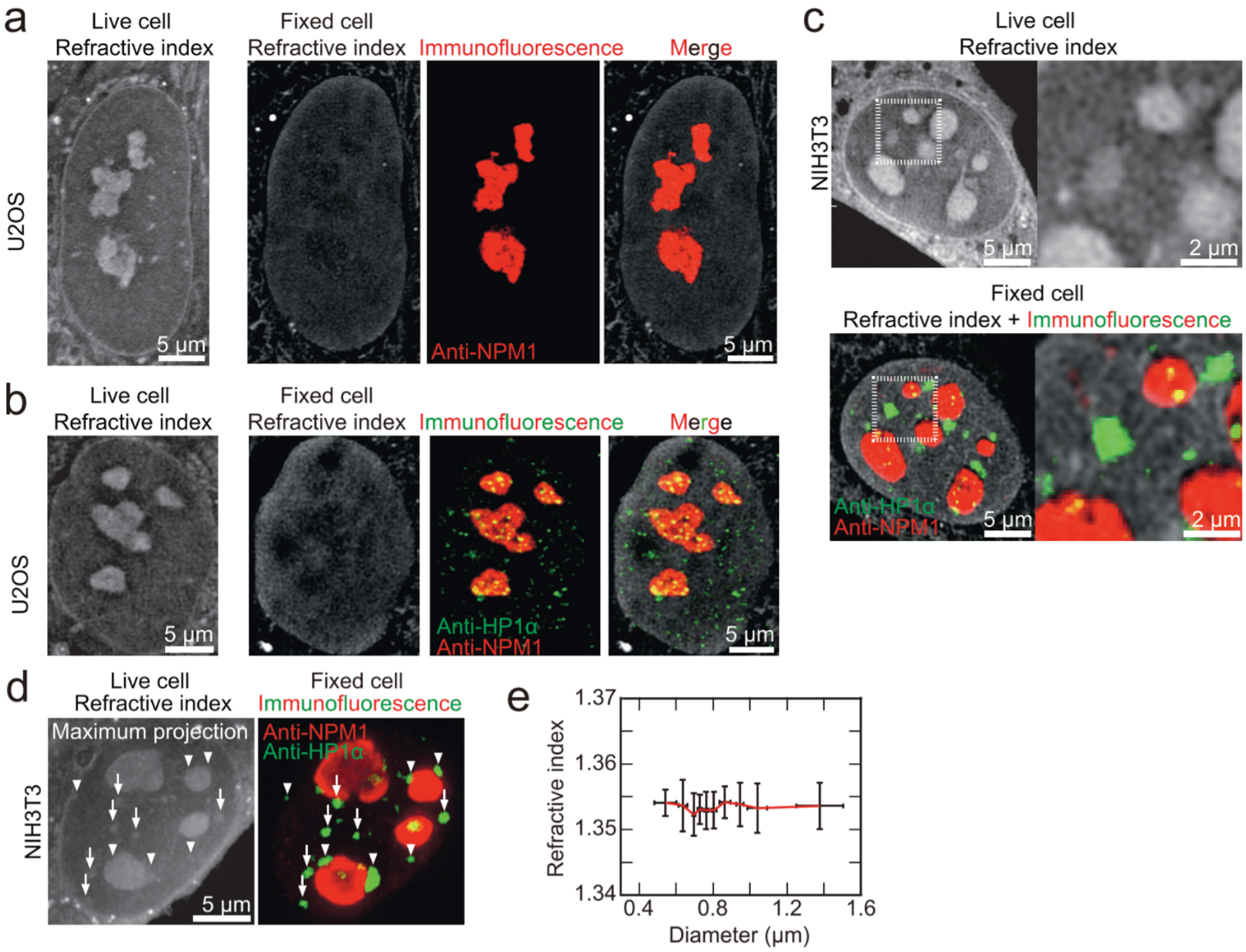

Fig S2. Refractive index imaging of U2OS and NIH3T3 cells before and after immunostaining with anti-NPM1 or anti-HP1α

a RI images of an intact live U2OS cell (left) and the same cell after immunostaining with anti-NPM1 (right). **b** RI images of an intact live U2OS cell (left) and the same cell after double-immunostaining with anti-NPM1 and anti-HP1α (right). **c** RI images of an intact live NIH3T3 cell (top) and the same cell after double-immunostaining with anti-NPM1 and anti-HP1α (bottom). The dashed boxes indicate areas for zoomed-in images on the right. **d** Maximum intensity projection of the 3D RI tomogram of an intact live NIH3T3 cell and immunofluorescence images after double-immunostaining with anti-NPM1 and anti-HP1α. Heterochromatin domains included in the RI quantifications are labeled with arrows, and those excluded are labeled with arrowheads (See Methods). **e** The distribution of intact refractive index values for individual heterochromatin in NIH3T3. n = 98. Error bars are std (8-10 condensates for each point). Mean values are connected with the red line. All refractive index images are adjusted to 1.337-1.37 except for (c) and (d), which are set to 1.34-1.37.

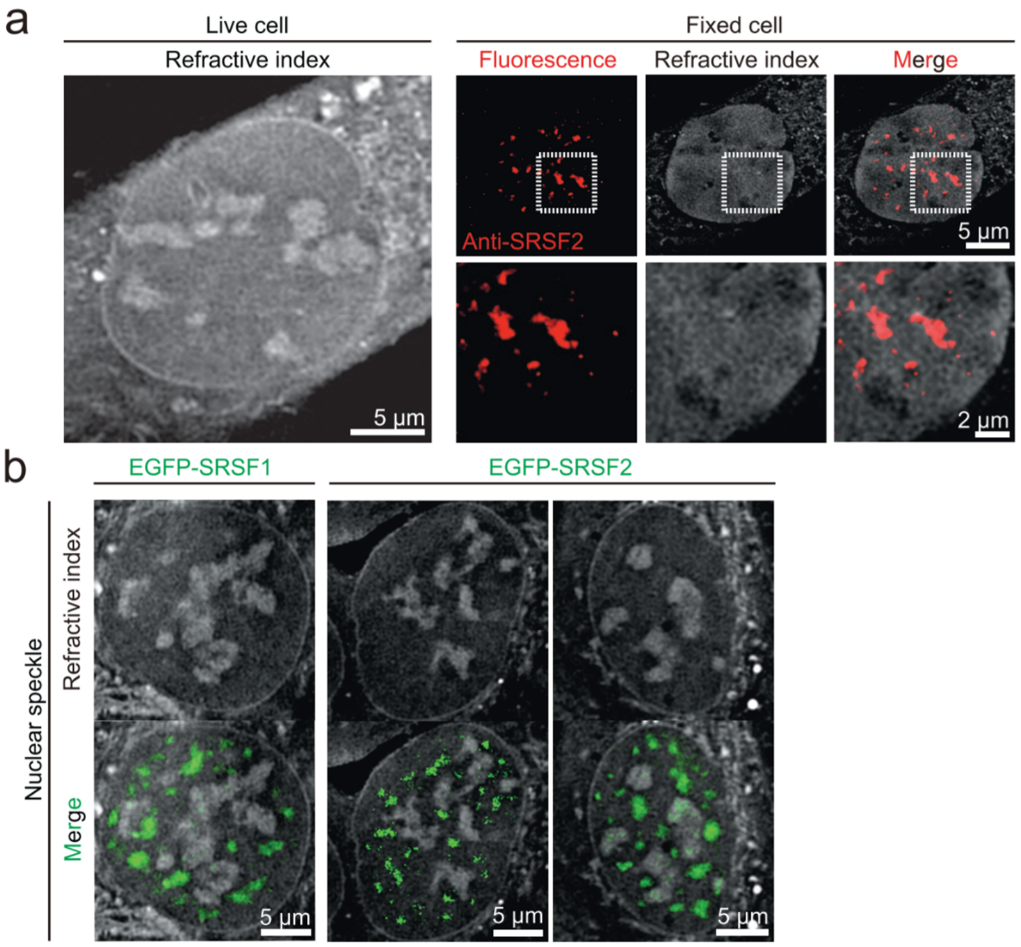

Fig. S3. Nuclear speckles are low-density condensates.

a (Left) RI image of an intact live U2OS cell. (Right top) RI and immunofluorescence images of the same cell after immunostaining with anti-SRSF2. The dashed boxes indicate areas for zoomed-in images at the bottom. **b** Combined RI and fluorescence images of live U2OS cells expressing EGFP-SRSF1 or EGFP-SRSF2. All refractive index images are adjusted to 1.337-1.37.

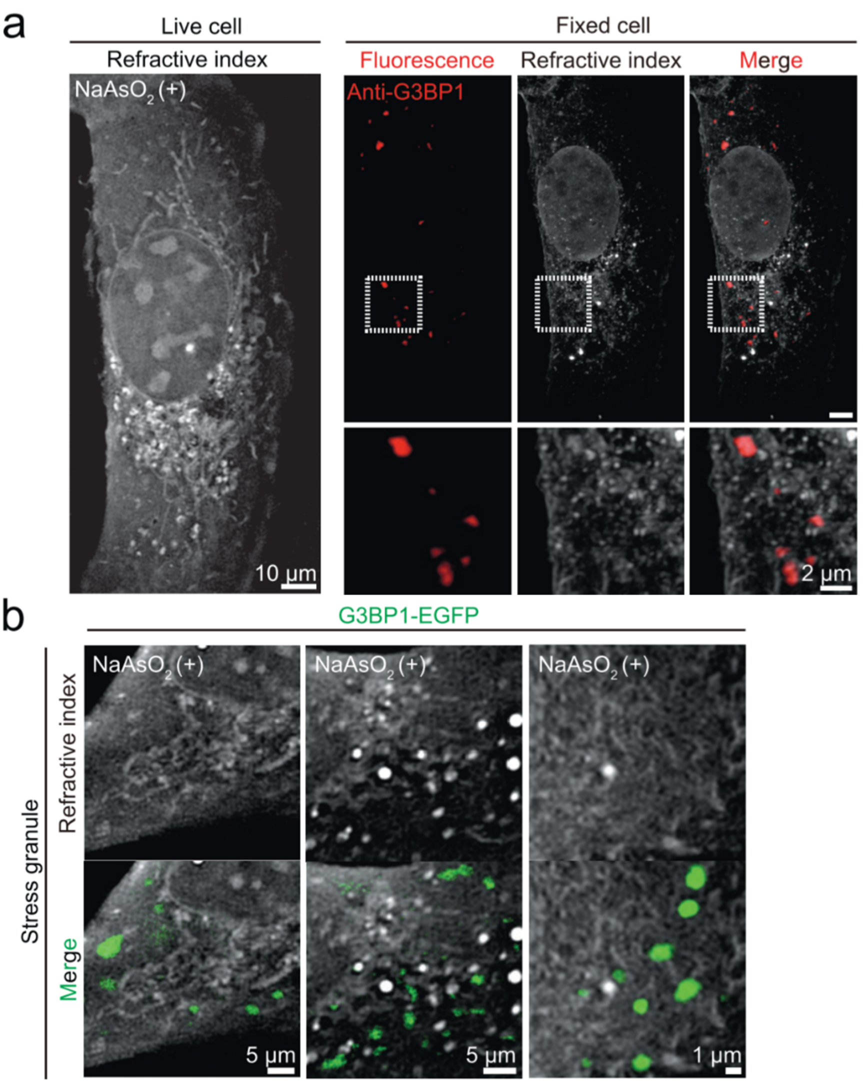

Fig S4. Stress granules are low-density condensates.

a (Left) RI image of an intact live U2OS cell. (Right top) RI and immunofluorescence images of the same cell after immunostaining with anti-G3BP1. The dashed boxes indicate areas for zoomed-in images at the bottom. **b** Combined RI and fluorescence images of live U2OS cells expressing G3BP1-EGFP treated with 500 μM sodium arsenite. All refractive index images are adjusted to 1.337-1.37 except for the image on the right in (**b**), which is set to 1.337-1.36.

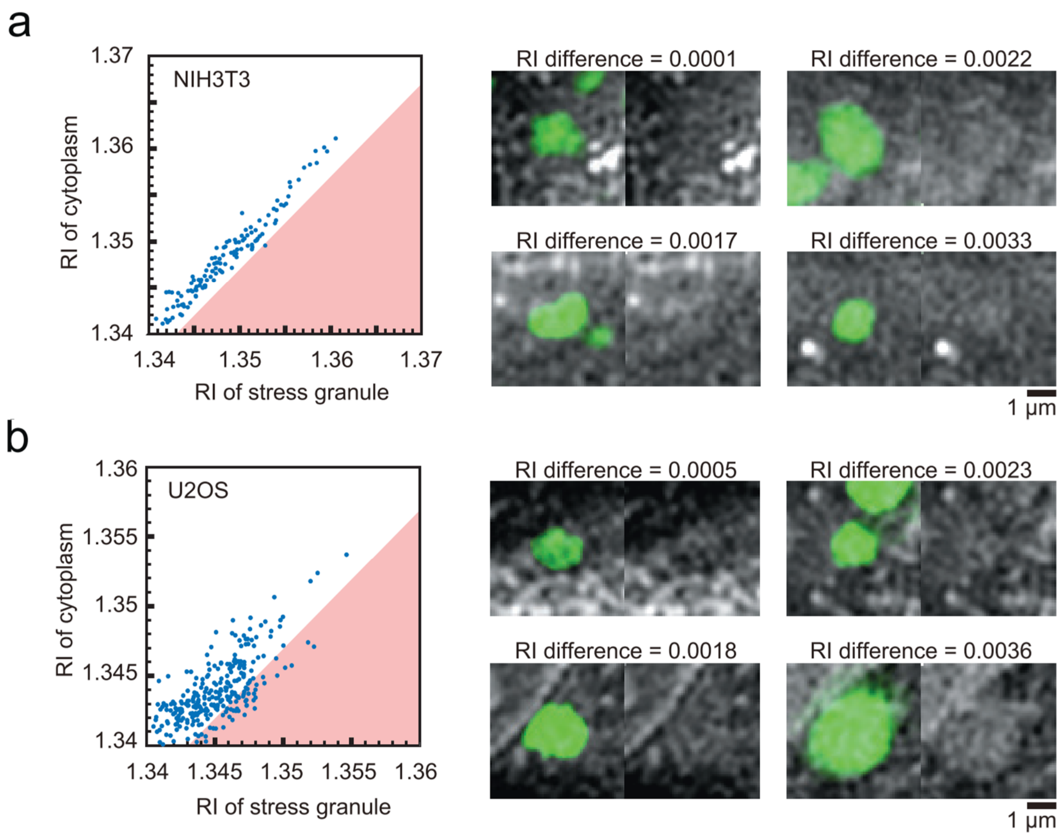

Fig S5. Comparison between refractive indices of stress granules and cytoplasm for NIH3T3 and U2OS cells.

a-b Scatter plot showing refractive indices of individual stress granules and surrounding cytoplasm for NIH3T3 (a) or U2OS cells (b) stably expressing G3BP1-EGFP. n = 120, 281, respectively. The colored areas indicate cases where the difference in the refractive index between the stress granule and the cytoplasm is greater than 0.003, and stress granules can be distinguished in RI images. Representative RI and fluorescence images are shown on the right.

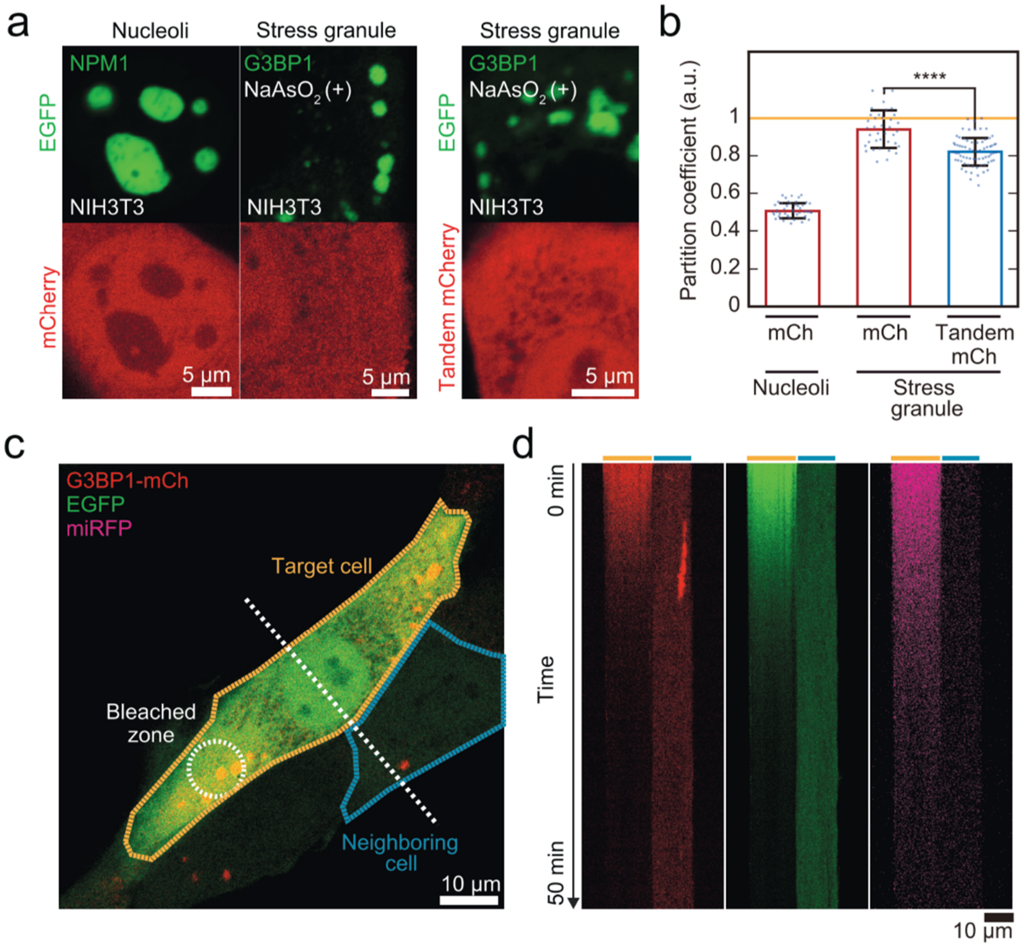

Fig S6. Probe partitioning and FLIP experiments characterize the internal organization of condensates.

a Fluorescence images of NIH3T3 cells stably expressing EGFP tagged NPM1 (left) or G3BP1 (middle and right). Cells are co-transduced with mCherry (left and middle) or tandem mCherry (right). G3BP1-EGFP expressing cells are treated with 500 μM sodium arsenite. **b** Partitioning coefficients of mCherry or tandem mCherry probes for condensates in (**a**). Data: center line = mean; whiskers = [mean + std, mean - std]; n = 46, 38, and 72, respectively. Distributions were statistically compared using the unpaired t-test. ****$p < 0.0001$. **c** To examine whether the bleaching effects are confined near the bleached zone, kymographs along the white dashed line are generated. **d** Kymographs showing fluorescence changes in each channel, generated along the white dashed line in (**c**). The fluorescence signals in the cell where the bleached zone is located decrease over time while those in the neighboring cell are stationary.

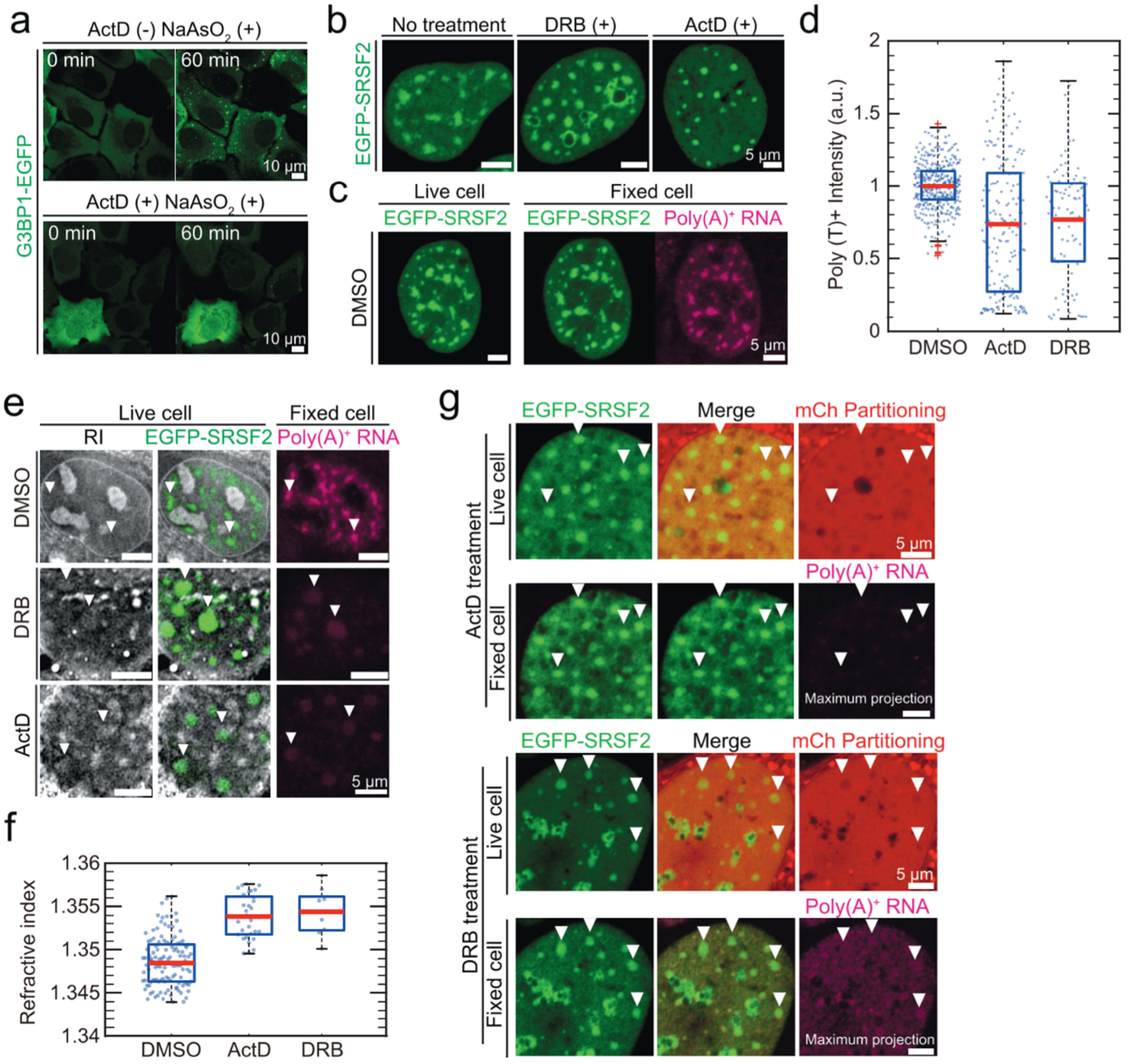

Fig S7. The effect of transcription inhibition-based RNA depletion on low-density condensates.

a Fluorescence images of U2OS cells stably expressing G3BP1-EGFP after treatment of sodium arsenite in the absence of ActD treatment (top) or in the presence of 12-hour pre-incubation with ActD (bottom). **b** Fluorescence images of U2OS cells stably expressing EGFP-SRSF2 in the absence of any treatment, 6 hours after DRB treatment, and 12 hours after ActD treatment. **c** Representative fluorescence and poly-dT RNA FISH images of an U2OS cell stably expressing EGFP-SRSF2 after DMSO treatment. **d** Distribution of poly-dT probe intensities in individual nuclear speckles of U2OS cells under DMSO, ActD, and DRB treatment. Poly-dT probe intensities are normalized with the average intensity of the DMSO-treated sample. n = 370 (DMSO), 175 (ActD), and 89 (DRB). **e** Combined RI (left) and fluorescence (middle) images of U2OS cells expressing EGFP-SRSF2 under DMSO (top), DRB (middle), and ActD (bottom) treatment. After fixation, the same cells were imaged for poly-dT RNA FISH (right). White arrowheads indicate examples of nuclear speckles invisible in the RI image of the DMSO-treated cell, yet visible in DRB- and ActD treated sample. **f** Quantification of refractive indices of individual nuclear speckles under DMSO, ActD, and DRB

treatment. For ActD/DRB-treated cells, only condensates exhibiting more than 20 % reduction in the poly-dT RNA signals were included. n = 121, 33, and 9 (DMSO-, ActD- and DRB-treated, respectively). **g** Representative images of mCherry probe partitioning for nuclear speckles after ActD- (top) or DRB treatment (bottom). White arrowheads indicate examples of nuclear speckles in which both probe exclusion and a decrease in the poly-dT signals were observed. RNA FISH images in (g) are in maximum intensity projection. Data in (d) and (f): center line = mean; box limits= [Q1, Q3]; whiskers = [Min Max].

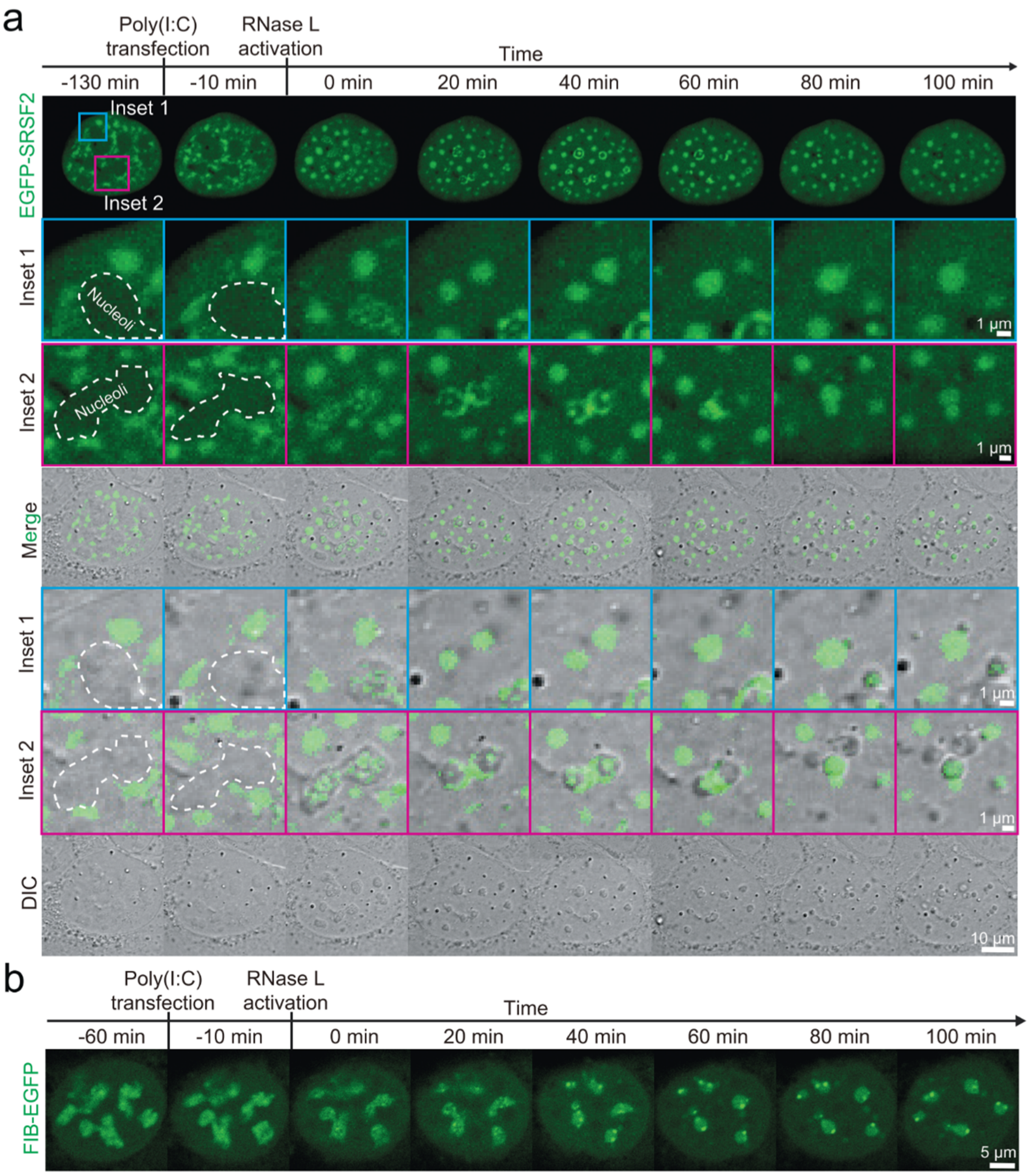

Fig S8. The effect of NLS-RNase L activation on nuclear speckles and nucleoli

a Time-lapse images of a U2OS cell stably expressing EGFP-SRSF2 and NLS-RNase L-P2A-BFP before and after poly(I:C) treatment. The chemical treatment was applied right after taking the first snapshot. Inset 1 and 2 highlight regions associated with nuclear speckles and nucleoli, respectively. Nucleoli outlines are indicated by the white-dashed curves. **b** Time-lapse images of a U2OS cell stably expressing EGFP-FIB and NLS-RNase L-P2A-BFP before and after poly(I:C) treatment.

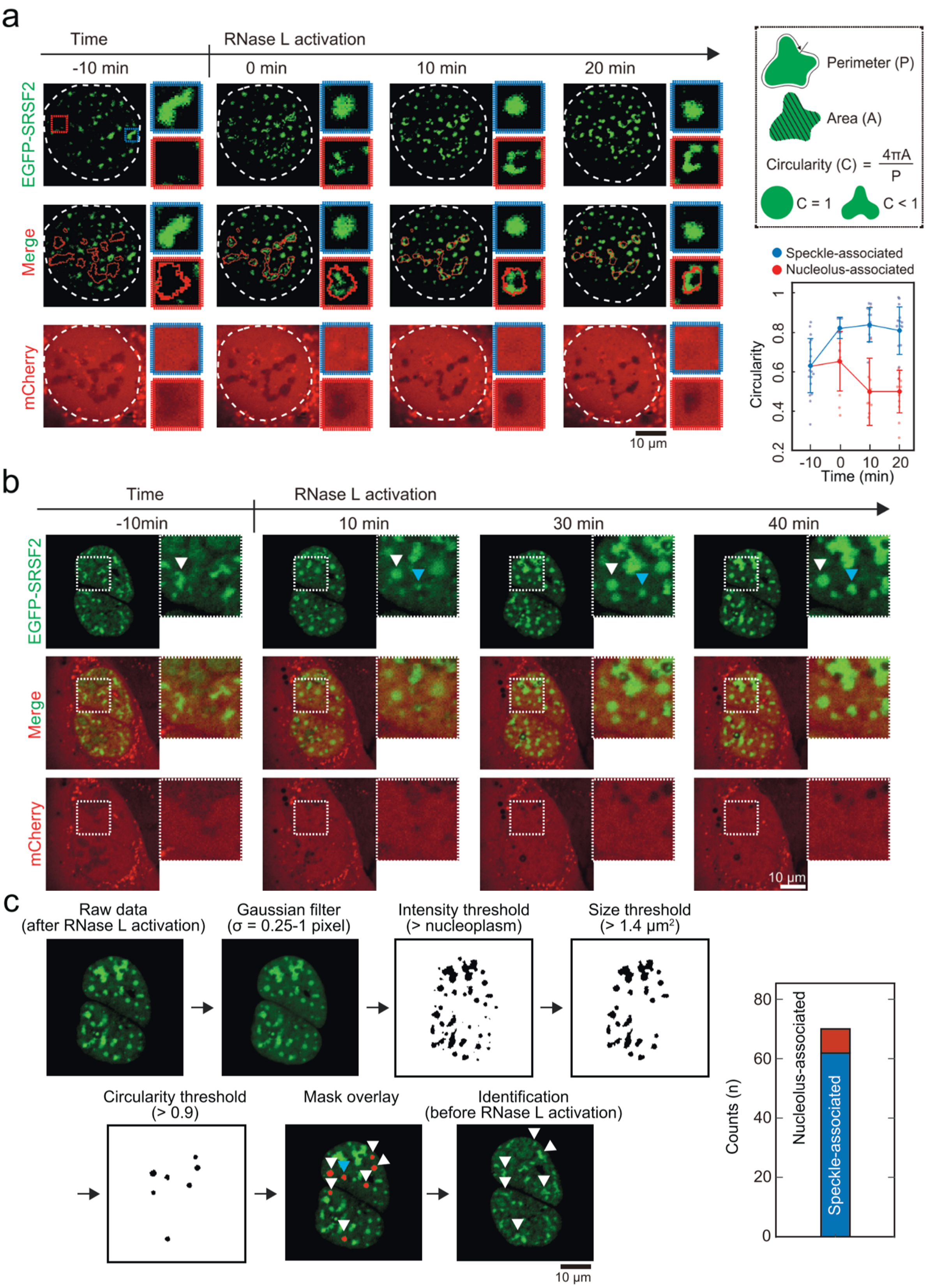
a
Time
RNase L activation
-10 min
0 min
10 min
20 min
EGFP-SRSF2
Merge
mCherry
10 μm
Perimeter (P)
Area (A)
Circularity (C) = 4πA / P
C = 1
C < 1
Speckle-associated
Nucleolus-associated
Circularity
Time (min)
b
Time
RNase L activation
-10min
10 min
30 min
40 min
EGFP-SRSF2
Merge
mCherry
10 μm
c
Raw data
(after RNase L activation)
Gaussian filter
(σ = 0.25-1 pixel)
Intensity threshold
(> nucleoplasm)
Size threshold
(> 1.4 μm²)
Circularity threshold
(> 0.9)
Mask overlay
Identification
(before RNase L activation)
10 μm
Counts (n)
Nucleolus-associated
Speckle-associated

Fig S9. Morphology changes of nuclear speckles under NLS-RNase L activation.

a (left)Time-lapse fluorescence images of a U2OS cell stably expressing EGFP-SRSF2, mCh probe, and NLS-RNase L-P2A-BFP before and after RNase L activation through poly(I:C) treatment. White dashed lines represent the nucleus boundary and red lines represent the boundaries of nucleoli obtained from intensity thresholding of mCh signals. The blue and red dashed boxes indicate the speckle- and nucleolus-associated SRSF2 foci, respectively. Time 0 is defined as a time point when the change in the nuclear speckle morphology begins to occur. (right; top) Schematics showing how the circularity of condensates is computed. (right; bottom) Temporal changes of the circularity of individual SRSF2-enriched condensates. After RNase L activation, a subpopulation of SRSF2 relocates into nucleoli (red). Center line = mean; whiskers = [mean + std, mean - std]. n = 19 (-10 min), 8 and 11 (0 min), 13 and 11 (10 min), and 12 and 11 (20 min). **b** Representative time-lapse fluorescence images of a U2OS cell stably expressing EGFP-SRSF2, mCh probe, and NLS-RNase L-P2A-BFP before and after RNase L activation. Zoomed-in images of the dashed area are shown on the right. Time is defined as in (a). **c** (left) The stepwise procedure for differentiating speckle-associated SRSF2 foci and nucleolus-associated ones in NLS-RNase L activated cells. First, a gaussian filter with a sigma of 0.25-1 pixel was applied to the raw data taken at 120-240 min after poly(I:C) treatment. Subsequently, three different types of thresholding were sequentially applied: intensity (EGFP signals higher than the nucleoplasm), size (greater than 1.4 μm^2), and circularity (higher than 0.9). To probe the validity, the procedure was applied for time-lapse images taken during RNase L activation, and the selected SRSF2 foci after thresholding were traced back to images prior to the activation. In this example, the procedure is applied to the cell in (b). The white arrowheads indicate speckle-associated SRSF2 foci, while the blue arrowheads indicate nucleolus-associated ones. (right) Out of 70 SRSF-positive foci, applying the differentiation procedure led to 61 foci tracked back to nuclear speckles.

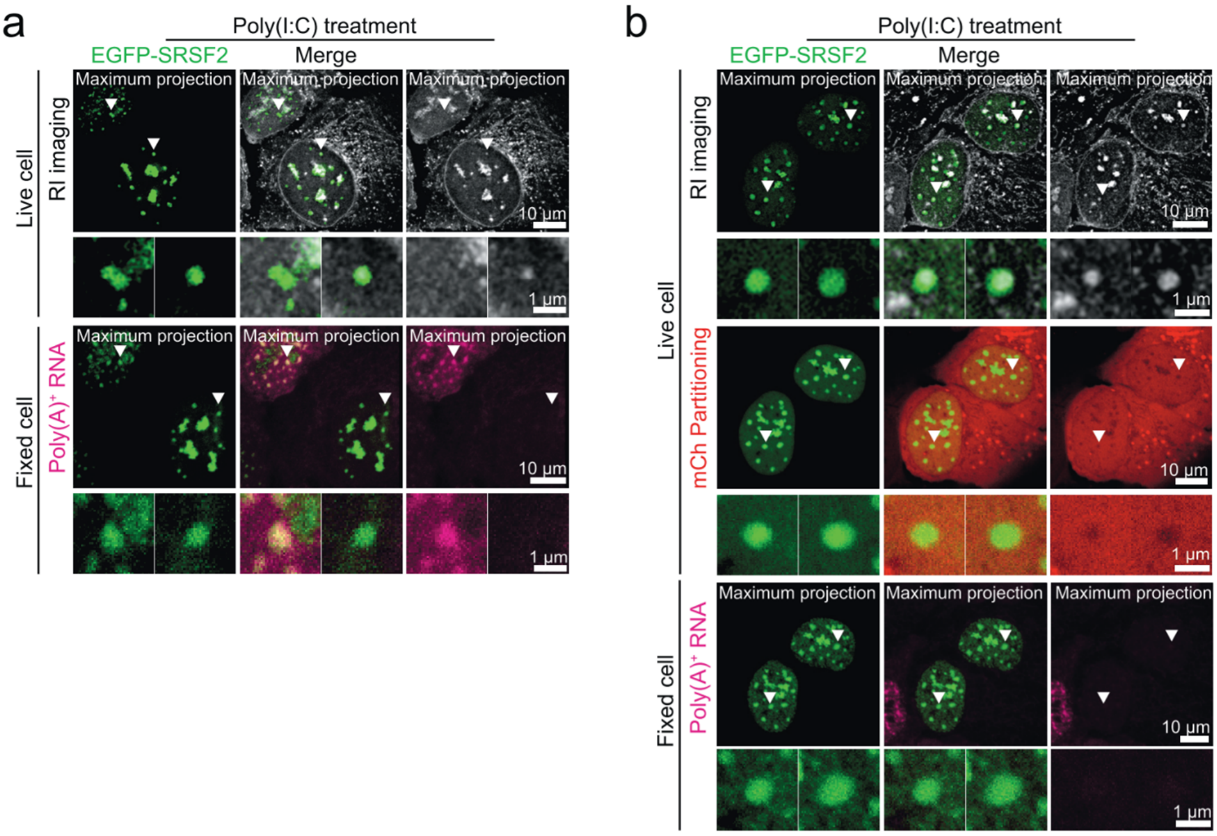

Fig S10. The effect of RNA depletion on the RI and probe partitioning of nuclear speckles.

a (top) Combined RI and fluorescence images of U2OS cells expressing EGFP-SRSF2 and NLS-RNase L-P2A-BFP after poly(I:C) treatment. After fixation, the same cells were imaged for poly-dT RNA FISH (bottom). **b** (top) Combined RI and fluorescence images of U2OS cells expressing EGFP-SRSF2, mCherry probe and NLS-RNase L-P2A-BFP after poly(I:C) treatment. After fixation, the same cells were imaged for poly-dT RNA FISH (bottom). For a-b, white arrowheads indicate regions for zoomed-in images below each panel. All RI and RNA FISH images are in maximum intensity projection. All RI images are adjusted to 1.34-1.36.

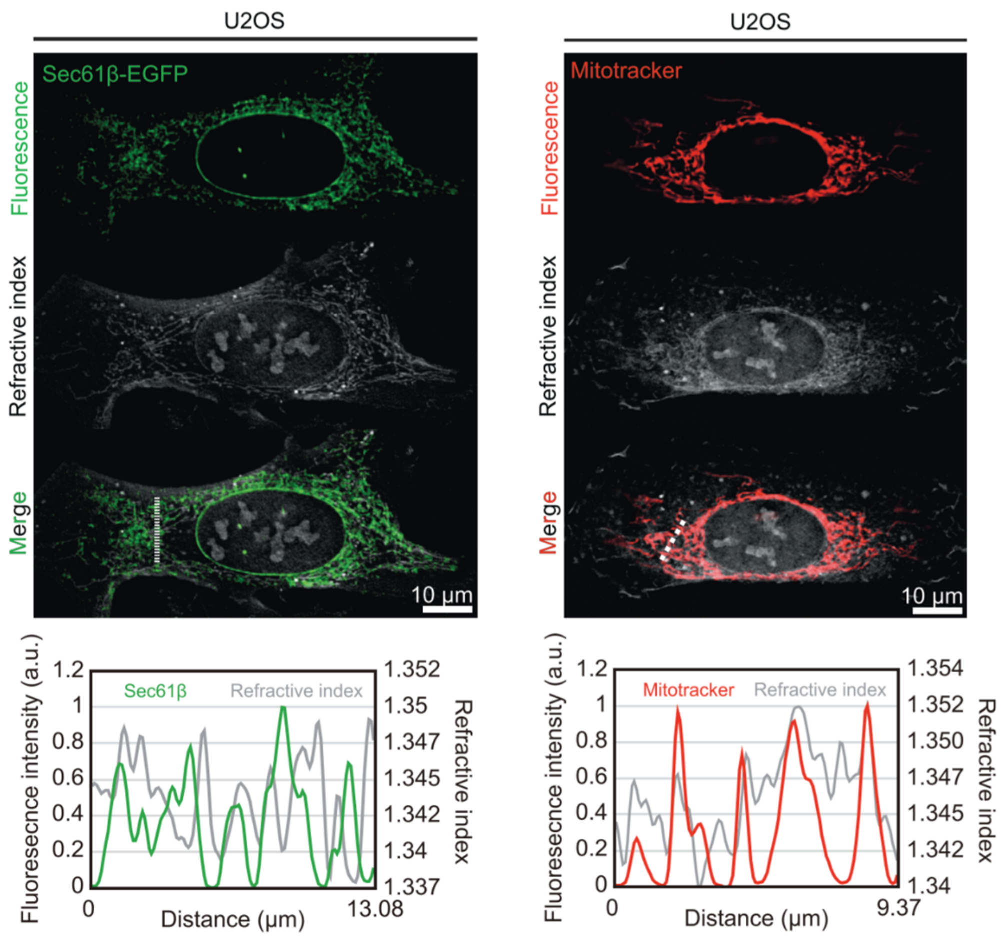

Fig S11. Cytoplasmic structures with high RI values correspond mostly to mitochondria.

(Left top) RI and fluorescence images of an U2OS cell expressing Sec61B-EGFP, an ER marker. (Left bottom) Combined profile plots of RI and fluorescence across the white dashed line. (Right top) RI and fluorescence images of an U2OS cell treated with the MitoTracker. (Right bottom) Combined profile plots of RI and fluorescence across the white dashed line. All refractive index images are adjusted to 1.34-1.37.
