# Supplementary material for: RNA-mediated demixing transition of low-density condensates
Source: Nat Commun. 2023 Apr 27;14:2425. doi: 10.1038/s41467-023-38118-z (PMC10140143; doi:10.1038/s41467-023-38118-z)
Supplement: Supplementary file 2 — Description of Additional Supplementary Files [file 41467_2023_38118_MOESM2_ESM.pdf]

File name: Supplementary Movie 1

Description: Time-lapse video of U2OS cells expressing EGFP-SRSF2. Cells were imaged with HT-2H. All refractive index images are adjusted to 1.337-1.37.

File name: Supplementary Movie 2

Description: Time-lapse video of U2OS cells expressing G3BP1-EGFP treated with 500 μM sodium arsenite. Cells were imaged with HT-2H. All refractive index images are adjusted to 1.337-1.37.
