# Supplementary material for: RNA-mediated demixing transition of low-density condensates
Source: Nat Commun. 2023 Apr 27;14:2425. doi: 10.1038/s41467-023-38118-z (PMC10140143; doi:10.1038/s41467-023-38118-z)
Supplement: Supplementary file 5 — Reporting Summary [file 41467_2023_38118_MOESM5_ESM.pdf]

Corresponding author(s): Yongdae Shin
Last updated by author(s): Mar 31, 2023

Reporting Summary

Nature Portfolio wishes to improve the reproducibility of the work that we publish. This form provides structure for consistency and transparency in reporting. For further information on Nature Portfolio policies, see our Editorial Policies and the Editorial Policy Checklist.

Statistics

For all statistical analyses, confirm that the following items are present in the figure legend, table legend, main text, or Methods section.

n/a	Confirmed	
☐	☒	The exact sample size (*n*) for each experimental group/condition, given as a discrete number and unit of measurement
☐	☒	A statement on whether measurements were taken from distinct samples or whether the same sample was measured repeatedly
☐	☒	The statistical test(s) used AND whether they are one- or two-sided<br>*Only common tests should be described solely by name; describe more complex techniques in the Methods section.*
☒	☐	A description of all covariates tested
☒	☐	A description of any assumptions or corrections, such as tests of normality and adjustment for multiple comparisons
☐	☒	A full description of the statistical parameters including central tendency (e.g. means) or other basic estimates (e.g. regression coefficient) AND variation (e.g. standard deviation) or associated estimates of uncertainty (e.g. confidence intervals)
☐	☒	For null hypothesis testing, the test statistic (e.g. *F*, *t*, *r*) with confidence intervals, effect sizes, degrees of freedom and *P* value noted<br>*Give P values as exact values whenever suitable.*
☒	☐	For Bayesian analysis, information on the choice of priors and Markov chain Monte Carlo settings
☒	☐	For hierarchical and complex designs, identification of the appropriate level for tests and full reporting of outcomes
☒	☐	Estimates of effect sizes (e.g. Cohen's *d*, Pearson's *r*), indicating how they were calculated

Our web collection on statistics for biologists contains articles on many of the points above.

Software and code

Policy information about availability of computer code

Data collection	All refractive index images were acquired with a commercial ODT system (HT-2H, Tomocube Inc., Republic of Korea). Fluorescence images were acquired using either HT-2H, or a Nikon A1 laser scanning confocal microscope. FLIP experiments were performed using Nikon A1 laser scanning confocal microscope.
Data analysis	The data analysis was performed with Excel (2016-Microsoft 365), ImageJ (version 1.53c-v), Tomostudio (version 3.0.3-3.3.9), and Matlab (R2020b-R2022b). Custom MATLAB code used in this study is available upon request.

For manuscripts utilizing custom algorithms or software that are central to the research but not yet described in published literature, software must be made available to editors and reviewers. We strongly encourage code deposition in a community repository (e.g. GitHub). See the Nature Portfolio guidelines for submitting code & software for further information.

Data

Policy information about availability of data

All manuscripts must include a data availability statement. This statement should provide the following information, where applicable:

- Accession codes, unique identifiers, or web links for publicly available datasets
- A description of any restrictions on data availability
- For clinical datasets or third party data, please ensure that the statement adheres to our policy

The data sets generated and/or analyzed during the current study are available from the corresponding author upon reasonable request.

Human research participants

Policy information about studies involving human research participants and Sex and Gender in Research.

Reporting on sex and gender	not applicable
Population characteristics	not applicable
Recruitment	not applicable
Ethics oversight	not applicable

Note that full information on the approval of the study protocol must also be provided in the manuscript.

Field-specific reporting

Please select the one below that is the best fit for your research. If you are not sure, read the appropriate sections before making your selection.

☒ Life sciences ☐ Behavioural & social sciences ☐ Ecological, evolutionary & environmental sciences

For a reference copy of the document with all sections, see nature.com/documents/nr-reporting-summary-flat.pdf

Life sciences study design

All studies must disclose on these points even when the disclosure is negative.

Sample size	No statistical methods were used to predetermine sample size. The sample size was determined by the condensates acquired and found to adequately sample the behaviors of condensates.
Data exclusions	The immuno-fluorescence labeling of fixed samples exhibited the common variability in staining quality, and poorly labeled cells could not be analyzed. Also, data from cells with fluorescence signals close to the background noise level were excluded from data analysis. For RNA depletion experiments, selection rules were applied to localize nuclear speckle-associated SRSF2 condensates (relevant details are provided in the Methods).
Replication	All experiments were performed independently more than three times. The same pattern was successfully reproduced in all trials.
Randomization	No animal or patient groups were used in our study, thus no randomization was required.
Blinding	Since all experiments used in this study did not require group allocations, blinding is not relevant for our study.

Reporting for specific materials, systems and methods

We require information from authors about some types of materials, experimental systems and methods used in many studies. Here, indicate whether each material, system or method listed is relevant to your study. If you are not sure if a list item applies to your research, read the appropriate section before selecting a response.

Materials & experimental systems

n/a	Involved in the study	
☐	☒	Antibodies
☐	☒	Eukaryotic cell lines
☒	☐	Palaeontology and archaeology
☒	☐	Animals and other organisms
☒	☐	Clinical data
☒	☐	Dual use research of concern

Methods

n/a	Involved in the study	
☒	☐	ChIP-seq
☒	☐	Flow cytometry
☒	☐	MRI-based neuroimaging

Antibodies

Antibodies used	For primary antibodies, mouse monoclonal Anti-SC35 (Abcam, ab11826, 1:200 dilution), mouse monoclonal Anti-NPM1 (Santa Cruz, sc-56622, 1:100 dilution), mouse monoclonal Anti-G3BP1 347 (Abcam, ab56574, 1:200 dilution), and rabbit monoclonal Anti-HP1α (Abcam, ab109028, 1:250 dilution) were used. For secondary antibodies, Anti-rabbit IgG conjugated with Alexa 488 (Invitrogen, A-11008, 1:500 dilution) and Anti-mouse IgG conjugated with Alexa 546 (Invitrogen, A-11030, 1:500 dilution) were used.

Validation	All antibodies were purchased from commercial sources and used according to the manufacturer's recommendations. Validation data were available in the data sheet provided by the manufacturers (Abcam, Santa Cruz, and Invitrogen).

Eukaryotic cell lines

Policy information about cell lines and Sex and Gender in Research

Cell line source(s)	Lenti-X 293T cells were purchased from Takara Bio (Cat# 632180). NIH 3T3 cells and U2OS cells were purchased from KCLB (Korean Cell Line Bank; Cat# 21658 and 30096).
Authentication	Cell lines used were not authenticated.
Mycoplasma contamination	The cells were not specifically tested mycoplasma contamination.
Commonly misidentified lines (See ICLAC register)	None of the cell lines used were listed in the ICLAC database
